# MUS81 cleaves TOP1-derived lesions and other DNA–protein cross-links

**DOI:** 10.1186/s12915-023-01614-1

**Published:** 2023-05-16

**Authors:** Victoria Marini, Fedor Nikulenkov, Pounami Samadder, Sissel Juul, Birgitta R. Knudsen, Lumir Krejci

**Affiliations:** 1 Department of Biology, Masaryk University, Kamenice 5/B07, Brno, 62500 Czech Republic; 2 International Clinical Research Center, Center for Biomolecular and Cellular Engineering, St. Anne’s University Hospital Brno, Pekařská 53, Brno, 60200 Czech Republic; 3 Department of Molecular Biology and Genetics, Aarhus University, Universitetsbyen 81, Aarhus, 8000 Denmark; 4 National Centre for Biomolecular Research, Masaryk University, Kamenice 5/C04, Brno, 625 00 Czech Republic

**Keywords:** DNA-protein cross-links repair, MUS81, TDP1, Topoisomerase 1

## Abstract

**Background:**

DNA-protein cross-links (DPCs) are one of the most deleterious DNA lesions, originating from various sources, including enzymatic activity. For instance, topoisomerases, which play a fundamental role in DNA metabolic processes such as replication and transcription, can be trapped and remain covalently bound to DNA in the presence of poisons or nearby DNA damage. Given the complexity of individual DPCs, numerous repair pathways have been described. The protein tyrosyl-DNA phosphodiesterase 1 (Tdp1) has been demonstrated to be responsible for removing topoisomerase 1 (Top1). Nevertheless, studies in budding yeast have indicated that alternative pathways involving Mus81, a structure-specific DNA endonuclease, could also remove Top1 and other DPCs.

**Results:**

This study shows that MUS81 can efficiently cleave various DNA substrates modified by fluorescein, streptavidin or proteolytically processed topoisomerase. Furthermore, the inability of MUS81 to cleave substrates bearing native TOP1 suggests that TOP1 must be either dislodged or partially degraded prior to MUS81 cleavage. We demonstrated that MUS81 could cleave a model DPC in nuclear extracts and that depletion of TDP1 in MUS81-KO cells induces sensitivity to the TOP1 poison camptothecin (CPT) and affects cell proliferation. This sensitivity is only partially suppressed by TOP1 depletion, indicating that other DPCs might require the MUS81 activity for cell proliferation.

**Conclusions:**

Our data indicate that MUS81 and TDP1 play independent roles in the repair of CPT-induced lesions, thus representing new therapeutic targets for cancer cell sensitisation in combination with TOP1 inhibitors.

**Supplementary Information:**

The online version contains supplementary material available at 10.1186/s12915-023-01614-1.

## Background

DNA-protein cross-links occur when a protein gets covalently bound to DNA. They represent toxic lesions that may interfere with replication and transcription progression [1]. DPCs can be formed by exogenous or endogenous agents and have non-enzymatic or enzymatic origins. Non-enzymatic DPCs occur when a protein is non-specifically cross-linked to the DNA and are frequently induced by chemicals such as cisplatin, endogenous reactive oxygen species or UV light. Enzymatic DPCs result from the activity of enzymes whose covalent reaction intermediates get trapped on DNA due to the presence of a poison or damaged bases in proximity [2–5]. Among these, the topoisomerases trapped on DNA belong to the most studied type of DPCs. Topoisomerases are essential enzymes in the DNA metabolism that release topological stress arising from the nature of the double-helical structure of DNA [6, 7]. These ubiquitous enzymes cleave DNA and form a transient covalent bond between a tyrosine in the active site and a phosphoryl group at the DNA break site, creating the so-called cleavage complex. This step is followed by the relaxation and religation of DNA with the concomitant liberation of the entire protein [7]. Human topoisomerase I (TOP1) cleaves only one DNA strand, to which it binds at the 3′ end [6]. A TOP1 cleavage complex (TOP1cc) may endure when occurring near a single-stranded break, near modified bases that impede DNA religation [8, 9] or in the presence of a TOP1 poison (such as camptothecin, CPT) [10]. Trapped TOP1ccs have been suggested to interfere with the replication or transcription, causing the arrest of the moving replication fork and leading to the formation of a double-strand break (DSB) [11–16]. The collision of the replication fork with TOP1cc triggers a DNA damage response and arrest of the cell cycle in the G2 phase [17]. Given the requirement of topoisomerase activities, Top1ccs, together with other DPCs, represent a serious threat to the cell. Several pathways have evolved to repair these lesions by targeting the DNA, the protein or the linking bond [1–5, 18].

One such pathway involves tyrosyl-DNA phosphodiesterase 1 (Tdp1), a protein that has been identified to be responsible for the removal of Top1ccs through hydrolysis of the 3′-phosphodiester bond [19–21]. Nevertheless, studies in *Saccharomyces cerevisiae* indicate that alternative repair pathways can also remove Top1 lesions since deletion of the *TDP1* gene does not induce higher sensitivity to CPT [22–25]. This was further supported by the observation that *RAD9* deletion sensitises *tdp1∆* strain to CPT [20, 22]. Similarly, the disruption of *RAD52* in yeast enhances the cytotoxicity of CPT [13, 26, 27], suggesting the generation of DSBs and a role of homologous recombination (HR) in the repair of Top1ccs. Recently, a protease Wss1/SPRTN-dependent repair pathway has also been identified as required for the repair of DPCs [25, 28–31]. Finally, several nucleases, including Mus81-Mms4, Mre11 and Rad1-Rad10, have been linked with the repair of Top1ccs [32–35]. However, their role in DPCs processing is still not well defined.

MUS81 is the catalytic subunit of the heterodimeric complexes MUS81-EME1 and MUS81-EME2 in humans and Mus81-Mms4 in budding yeast (for simplicity, referred to as MUS81-EME1, MUS81-EME2 and Mus81-Mms4, respectively). These proteins belong to the XPF/MUS81 protein family, which plays an important role in DNA repair. All MUS81 complexes possess structure-specific endonuclease activity and preferentially cleave branched DNA substrates, including 3′ flap, replication fork and nicked Holliday junction in vitro [36–39]. The MUS81 protein has been shown to play a role in the resolution of recombinant intermediates and replication fork stability [27, 40–43] and has been implicated in the repair of mitomycin C- or cisplatin-induced cross-links [44, 45].

In this study, we aimed to test the ability of Mus81-Mms4, MUS81-EME1 and MUS81-EME2 complexes to cleave DNA-protein cross-links, particularly those bearing covalently bound TOP1. To this end, we prepared a series of DNA substrates that mimic a DNA-protein lesion as well as substrates covalently linked to TOP1. Our biochemical data suggest that MUS81 can remove TOP1, but similarly to TDP1, it requires the proteolytic degradation of TOP1 within the cleavage complex to achieve this process. Moreover, the simultaneous depletion of MUS81 and TDP1 in cells suggests that MUS81 plays a backup role to TDP1 in the DNA damage response. Importantly, since compounds inducing TOP1-mediated DPCs are currently used in cancer treatment [46–48], understanding the underlying mechanism of the repair pathway is essential for improving the efficiency of combination therapy with TDP1 and MUS81 inhibitors.

## Results

### Mus81-Mms4 and MUS81-EME1 cleave various fluorescein-modified substrates

**Fig. 1:**
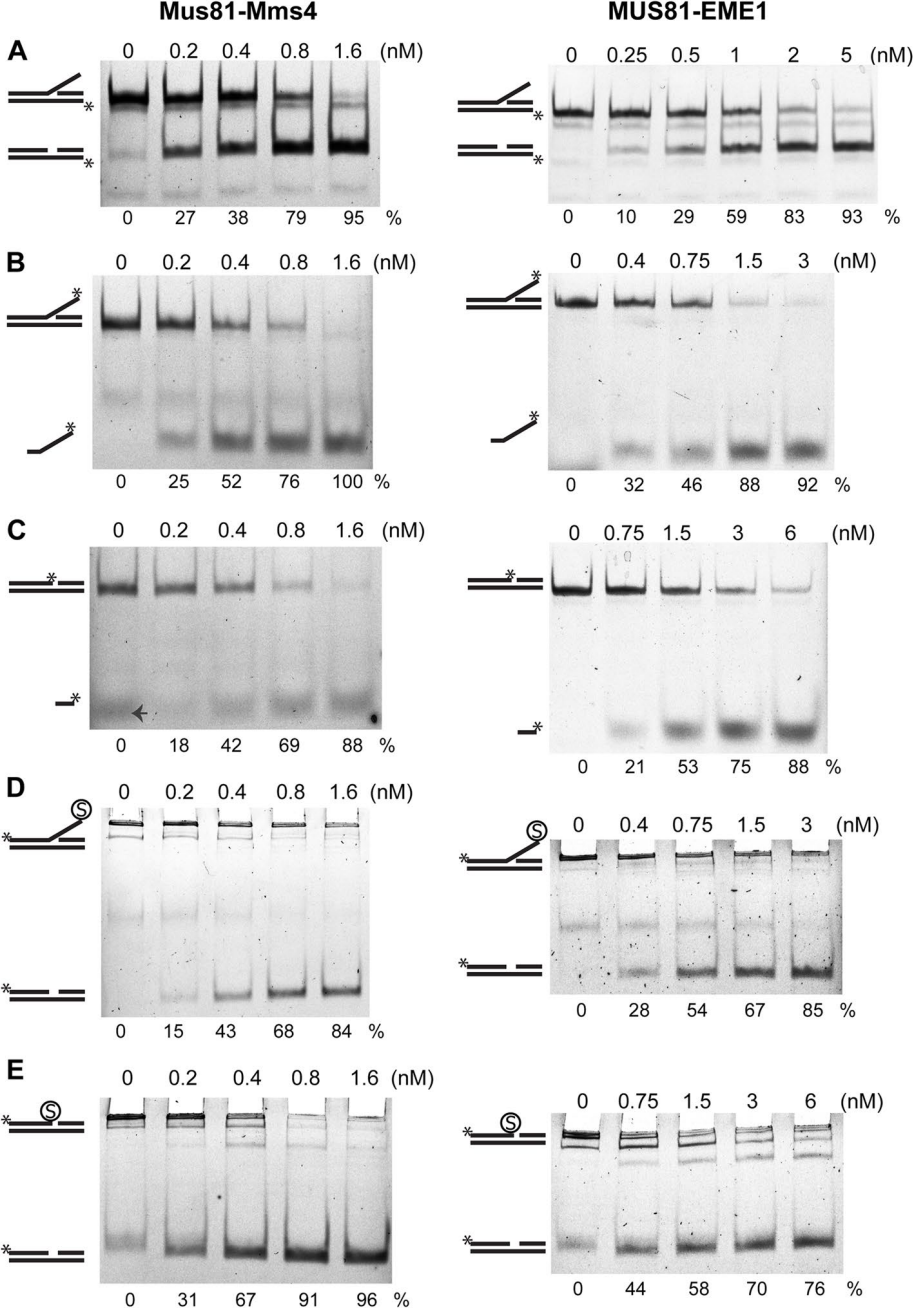
Mus81-Mms4 and MUS81-EME1 cleave fluorescein and streptavidin substrates. The nuclease activity assay for Mus81-Mms4 and MUS81-EME1 was performed by mixing the DNA substrate with the indicated protein complex concentrations and incubating for 15 min at 30 °C or 37 °C, respectively. The samples were resolved by native PAGE. The asterisk marks the position of fluorescein. Mus81-Mms4 and MUS81-EME1 efficiently cleave standard 3′ flap substrate (**A**), nicked duplex and 3′ flap substrates containing the fluorescein label at the 3′ end of the cleaved strand (**B**, **C**), and nicked duplex and 3′ flap with streptavidin (S) attached to the 3′ end of the cleaved strand (**D**, **E**). Numbers under the gel pictures represent the percentage of cleavage product calculated from the sum of substrate and product band intensities, except for the reaction with the streptavidin substrates, where the substrate band intensity of the control lane was taken as 100%. A small arrow (**C**) indicates Orange G containing the loading buffer used in that particular lane for gel migration control

To test whether Mus81-Mms4 and MUS81-EME1 can cleave substrates with a DPC lesion, we first created substrates modified only with fluorescein. The fluorescein molecule also serves to mimic the tyrosine group of TOP1 active site [49]. The nuclease activity assay was performed with two substrates, a 3′ flap with fluorescein attached at the 3′ end of the single-stranded flap and a nicked duplex with fluorescein at the 3′ end of the nick (Additional file 1: Fig. S1A). The DNA substrates were incubated with increasing amounts of Mus81-Mms4 or MUS81-EME1, and the reaction mixtures were analysed by native PAGE. As shown in Fig. 1B, C, both Mus81-Mms4 and MUS81-EME1 were fully capable of cleaving these substrates with comparable efficiency to the 3′ flap substrate labelled at the bottom strand (Additional file 1: Fig. S1B and Fig. 1A). These data suggest that fluorescein at the 3′ end of a flap or nick, mimicking a tyrosine residue, does not represent an obstacle to the nuclease activity of these enzymes.

To verify that the activity we observe with the nicked duplex is specific to MUS81, we compared wild-type (WT) and a nuclease-dead (ND) mutant of MUS81. For this purpose, we used a truncated version of the complex, MUS81(246-551)-EME1(178-570) [50] and ND mutant containing D338A and D339A mutations [51]. To better monitor the cleavage, the nicked duplex was labelled with two fluorescent dyes: fluorescein and CY5 at the 3′ end of the nick and the 5′ end of the bottom strand, respectively (Additional file 2: Fig. S2A). While incubation of the WT complex cleaved the substrates in a concentration-dependent manner, no products were obtained with the ND complex (Additional file 2: Fig. S2A). These data confirm the intrinsic MUS81 nuclease activity requirement for cleavage of the nicked duplex. Next, we checked the cleavage position of MUS81-EME1 within the nicked duplex, using two substrates with fluorescein either at the 3′ end of the nick or at the 5′ end of the same oligonucleotide together with CY5 label at the 5′ end of the bottom strand (Additional file 2: Fig. S2B). The incubation of the MUS81-EME1 complex with these substrates showed no cleavage of the bottom strand and endonucleolytic processing of the 3′ end of the nick. This cleavage is similar to the one observed for Mus81-Mms4 complex [37].

### Streptavidin does not prevent cleavage by Mus81-Mms4 or MUS81-EME1

To further analyse the results obtained with the fluorescein modification, we tested the effect of a bulkier modification compared with that of a sole tyrosine residue. Therefore, we designed substrates containing a biotin group to which streptavidin could be attached. Two streptavidin-bound substrates, a 3′ flap and a nicked duplex (Additional file 1: Fig. S1C), were incubated with increasing amounts of Mus81-Mms4 or MUS81-EME1. The reaction mixtures were analysed as mentioned above. Both substrates were very efficiently cleaved by both Mus81-Mms4 and MUS81-EME1 (Fig. 1D, E). Although the substrate containing streptavidin remained very close to the gel wells, the cleaved substrate could run further into the gel. Based on this result, we conclude that even a 60-kDa bulky protein does not block the nucleolytic cleavage of the substrate by the tested nuclease complexes.

### Native TOP1 prevents cleavage

**Fig. 2:**
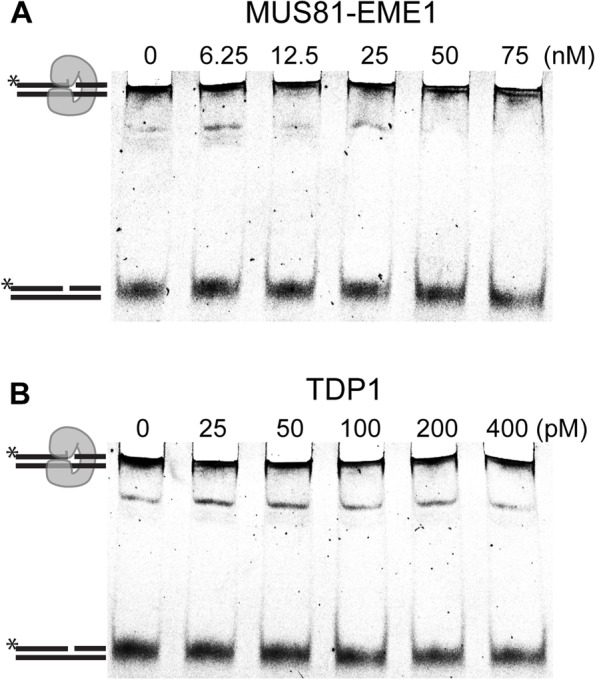
MUS81-EME1 and TDP1 cannot remove TOP1 in a native state. The substrate bearing native TOP1 covalently bound to the 3′ end of the nick was mixed with the indicated amounts of MUS81-EME1 or TDP1, and the mixture was incubated for 15 min at 37 °C for MUS81-EME1 or at 30 °C for TDP1. Neither MUS81-EME1 (**A**) nor TDP1 (**B**) can cleave the native TOP1 substrate. The reaction was stopped with SDS in both cases, and the samples were run on native PAGE

While streptavidin attached to DNA through the biotin group may not interact with the DNA itself, TOP1 is known to embrace DNA (PDB 1A31 [52]); PDB 1K4S [53]). To test whether this affects the nuclease activity, we used a suicide substrate, with TOP1 covalently bound to a nicked duplex and in the native state as previously described (Additional file 1: Fig. S1D) [54, 55]. The substrate was incubated with increasing amounts of MUS81-EME1 or TDP1, and the samples were loaded on native PAGE. However, we did not observe any cleavage by MUS81-EME1 (Fig. 2A) nor by TDP1 (Fig. 2B), suggesting that native TOP1 makes the cleavage site inaccessible for cleavage by MUS81-EME1 or TDP1.

### TDP1 and MUS81 activity requires the proteolytical processing of TOP1-conjugated substrates

It has been proposed that TOP1 must undergo proteolysis to facilitate the removal of the resulting peptide from DNA by TDP1 [56]. Thus, we reasoned that the MUS81 endonucleases would also cope with the trapped TOP1 after its processing. To address this, we prepared suicide substrates containing the covalently bound TOP1 and treated them with trypsin (“Methods”, Additional file 3: Fig. S3 and Additional file 4: Supplementary Methods). Using this procedure, we designed three substrates, as depicted in Additional file 1: Fig. S1E. The slower-migrating band corresponds to the substrate cleaved by TOP1 and bears the remaining peptide (oligo 7^−^ + TOP1 peptide). The faster band corresponds to the unmodified DNA substrate (oligo 7) due to not fully efficient DNA cleavage by TOP1 (Additional file 3: Fig. S3B and C).

**Fig. 3:**
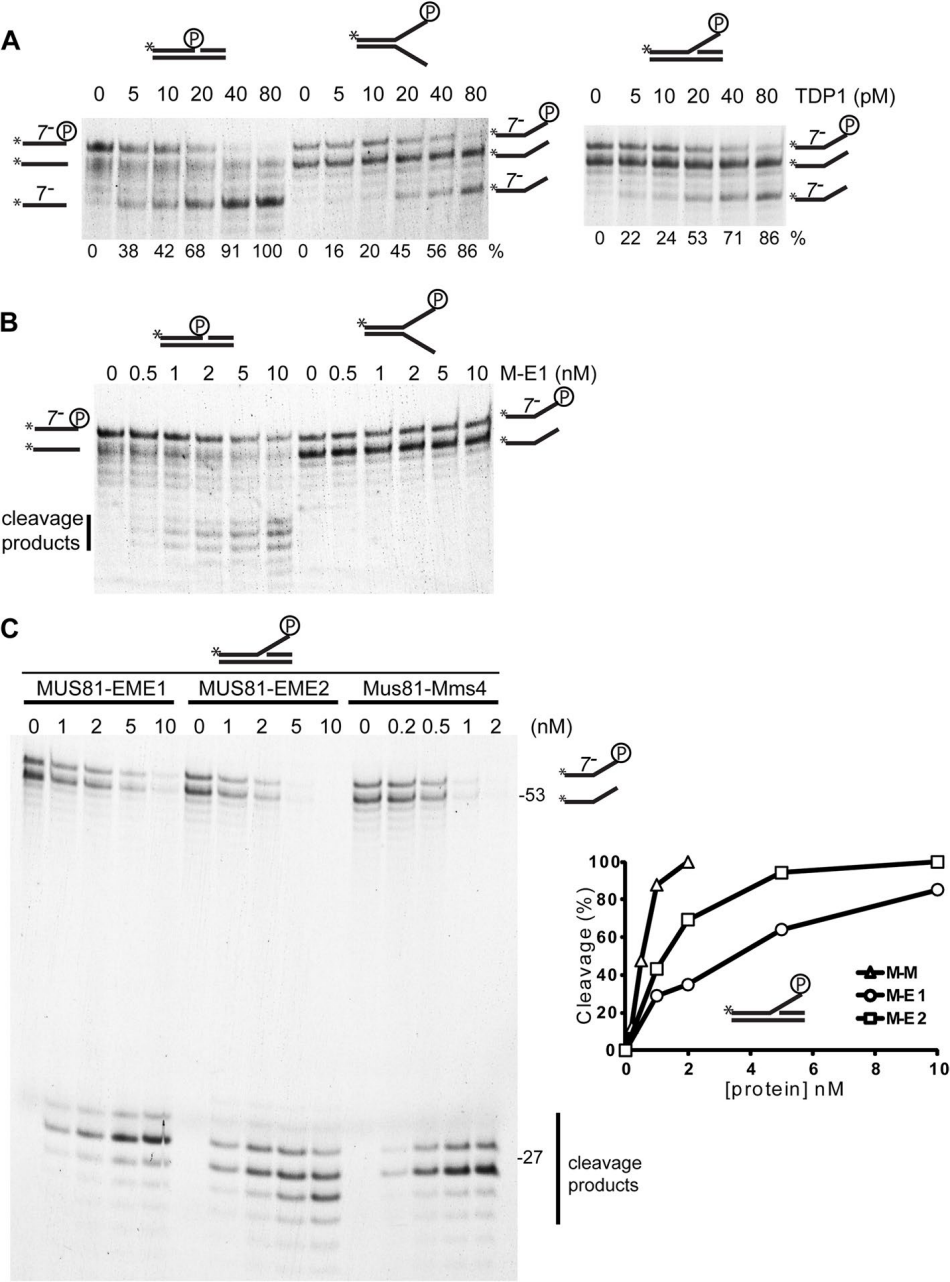
Trypsinised DNA substrates and their cleavage by TDP1 and MUS81 complexes. DNA substrates bearing a small TOP1 peptide were prepared by treating TOP1-linked DNA with trypsin. **A** TDP1 removes the TOP1 peptide from nicked duplex, Y-form and 3′ flap. The trypsinised substrates were incubated with TDP1 at 30 °C for 15 min. The reaction was stopped with SDS and resolved by denaturing PAGE. Numbers under the gel pictures represent the percentage of cleavage product calculated from the sum of the modified substrate and product band intensities. **B** MUS81-EME1 (M-E1) cleaves trypsinised nicked duplex in contrast to Y-form DNA. **C** Mus81-Mms4 (M-M), as well as MUS81-EME1 (M-E1) and MUS81-EME2 (M-E2), efficiently cleave the trypsinised 3′ flap substrate (left). Quantification of the activity (right). Only the bands corresponding to the oligo 7^−^ bearing the small TOP1 peptide were quantified. The trypsinised substrates were incubated with increasing amounts of MUS81 complexes at 37 °C for 15 min. The reaction was stopped by adding SDS and resolved by denaturing PAGE

The above-described substrates were incubated with TDP1 or MUS81-EME1 proteins, and the samples were analysed by denaturing PAGE. As expected, TDP1 was able to remove the TOP1 peptide very efficiently from all three modified substrates while leaving the unmodified substrates intact, indicating the specificity of the reaction toward modified DNA (Fig. 3A). MUS81-EME1 is also able to entirely cleave the nicked duplex (Fig. 3B) and the trypsinised 3′ flap (Fig. 3C) with similar efficiency as for unmodified substrates (note the disappearance of both the upper and lower bands of the substrate, Additional file 5: Fig. S4A). Because the Y-form substrate is not suitable for MUS81 endonucleases, we also did not observe any cleavage of the trypsinised Y-form (Fig. 3B).

To assess whether this activity is conserved, we also performed the assays with Mus81-Mms4 and MUS81-EME2. As shown in Fig. 3C, all complexes were able to fully cleave the trypsinised 3′ flap with similar efficiency as the unmodified substrate (Fig. 3C and Additional file 5: Fig. S4). Moreover, we observed that Mus81-Mms4 and MUS81-EME2 were even more efficient nucleases than MUS81-EME1 (Fig. 3C), in accordance with previously published data [39].

### MUS81 complex cleaves streptavidin-modified nicked duplex in human nuclear extracts

**Fig. 4:**
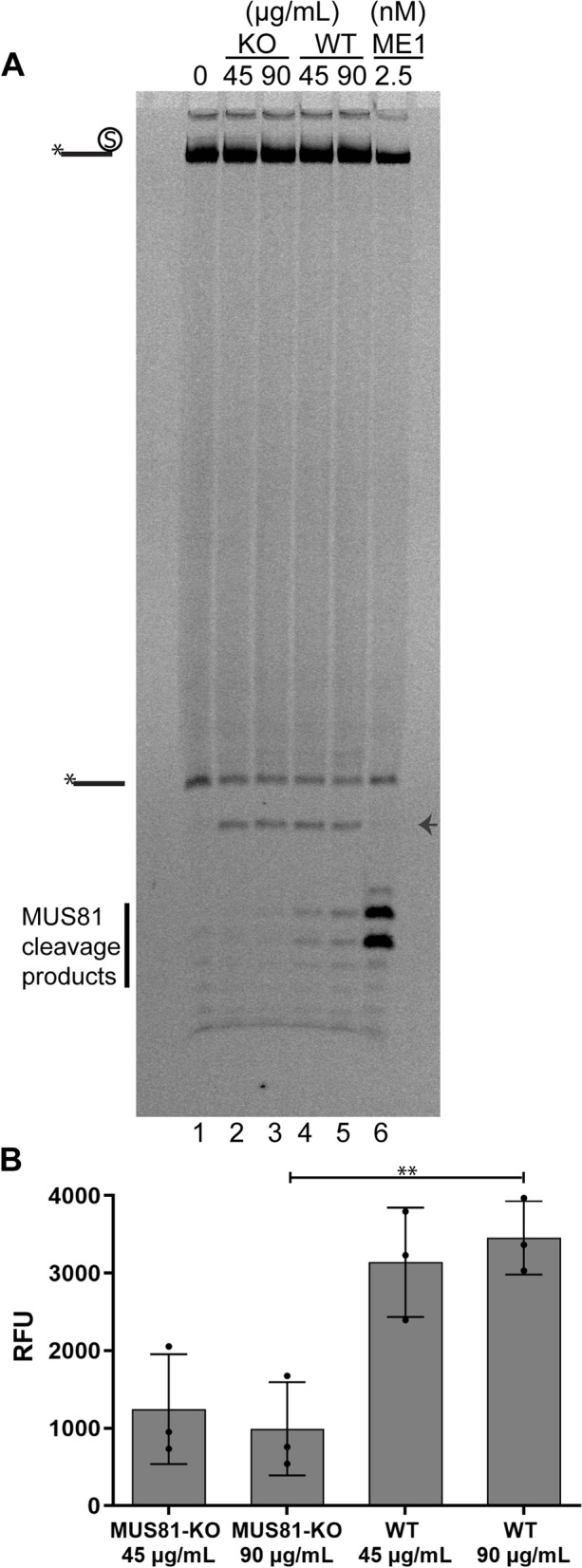
Cleavage of DPCs by MUS81 in nuclear extracts. **A** A nicked duplex modified with streptavidin was incubated with different amounts of nuclear extracts from CAL51 MUS81-KO (lanes 2 and 3) or WT CAL51 (lanes 4 and 5) cells at 37 °C for 1 h. The reaction was also performed with recombinant MUS81-EME1 complex (2.5 nM, lane 6). The small arrow indicates an unspecific band, most likely the product of the removal of the biotin moiety from the residual oligonucleotide without streptavidin. **B** Quantification of the reaction products from **A**. Relative fluorescence units (RFU) are the arbitrary units of the imaging system for fluorescence intensity. The means and standard deviations from three independent experiments are shown. The *P* value (**P < 0.01) was calculated through an unpaired two-tailed *t*-test. Individual data values can be found in Additional file 10

Our biochemical analysis shows that MUS81 endonucleases may play a role in the repair of TOP1 lesions and other DPCs. To address this role in cells, we tested the processing of DPC-like substrates using nuclear cell extracts. To this end, we generated a MUS81 knockout CAL51 cell line using CRISPR/Cas9 and confirmed the loss of MUS81 protein expression (Additional file 6: Fig. S5A). Next, we prepared nuclear extracts from CAL51 WT and MUS81-KO cell lines and incubated them with a DPC-mimicking substrate, i.e. nicked duplex-bearing streptavidin at the 3′ end of the nick (Additional file 1: Fig. S1F). We included phosphorothioate bonds to protect the other free ends of the substrate from exonuclease degradation. We observed distinguishable bands in CAL51 WT extracts that matched cleavage sites of this DPC substrate by recombinant MUS81-EME1 complex (Fig. 4A, lanes 4–6). Accordingly, these bands reduced to background signal in the CAL51 MUS81-KO extract sample (Fig. 4A, lanes 2 and 3 and Fig. 4B), indicating that MUS81 also cleaves such a model DPC in a cell-free setting.

### MUS81 represents an alternative pathway to TDP1

**Fig. 5:**
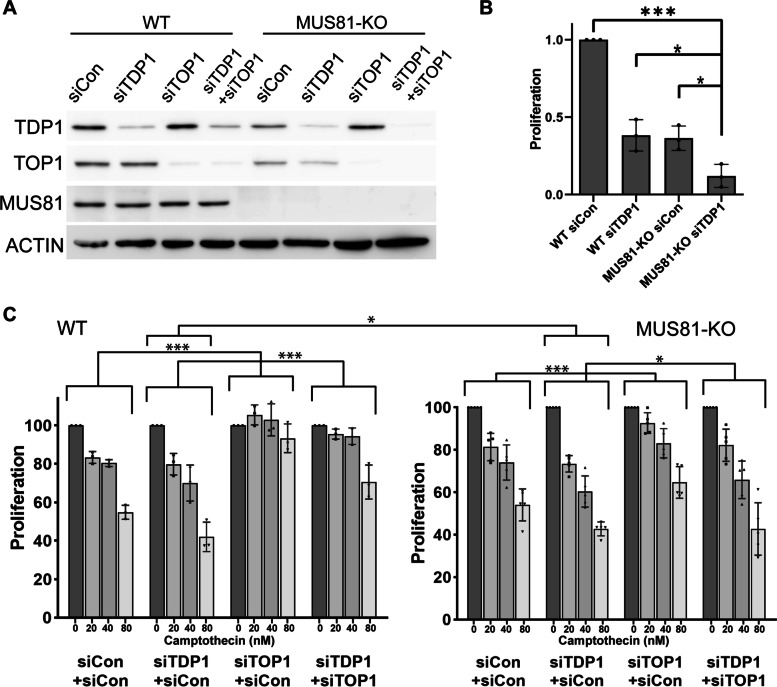
Depletion of TDP1 sensitises MUS81-KO cells to CPT and affects their proliferation. **A** The efficiency of TDP1 and TOP1 knockdown in CAL51 WT and MUS81-KO cells was determined by Western blot. Actin was used as a loading reference. **B** The proliferation of untreated CAL51 WT and MUS81-KO cells with or without TDP1 depletion was measured by WST-1 assay. The means and standard deviations from three independent experiments are shown. The *P* values were calculated through an unpaired two-tailed *t*-test (*P < 0.05, ***P < 0.001). Individual data values can be found in Additional file 10. **C** Cell proliferation of CAL51 WT and MUS81-KO cells simultaneously treated with corresponding siRNA (siCon, siTDP1, or siTOP1) and the presence of increasing CPT concentrations. To maintain the amount of siRNA of the double mutant in all samples, extra siCon was added for single-depleted and control cells. The means and standard deviations from three (WT) and five (KO) independent experiments are shown. The statistical significance was determined by two-way ANOVA test (*P < 0.05, ***P < 0.001). Individual data values can be found in Additional file 10

To confirm the role of the human MUS81 in processing DPCs and its relationship with TDP1 in cells, we examined the sensitivity of cells to low doses of CPT in the presence or absence of these two proteins. Using specific siRNA, we depleted TDP1 in CAL51 WT and MUS81-KO cells (Fig. 5A) and assessed cell proliferation by the WST-1 assay. While at given conditions, loss of MUS81 or TDP1 alone had no significant effect on the cells’ sensitivity, their combination showed CPT concentration-dependent sensitisation (Additional file 6: Fig. S5B), indicating that both TDP1 and MUS81 are involved in the processing of TOP1-mediated DPCs.

Furthermore, we observed that even without CPT treatment, CAL51 MUS81-KO cells and TDP1 downregulated CAL51 cells showed slower proliferation (Fig. 5B). Interestingly, the depletion of TDP1 in CAL51 MUS81-KO cells led to an additional decrease in cell proliferation, further supporting the role of TDP1 and MUS81 in separate pathways.

When treating CAL51 WT cells with higher CPT concentrations, we observed a reduction in proliferation, which is almost completely reversed after TOP1 depletion (Fig. 5C). Depletion of TDP1 exacerbates the effect of CPT and is also rescued by the simultaneous co-depletion of TOP1, even though to a lesser extent than WT cells. Next, we performed the same experiments in CAL51 MUS81-KO cells and cells co-depleted for TDP1 (Fig. 5C). In contrast to MUS81-KO cells, the proliferation of TDP1/MUS81 double deficient cells was only slightly improved upon depletion of TOP1, indicating a possible role of MUS81 and TDP1 in the repair of TOP1-independent lesions.

## Discussion

Genetic experiments in budding yeast have shown the existence of TOP1cc repair pathways involving various nucleases, including Mre11, Rad1-Rad10 or Mus81-Mms4 [22, 32, 33]. Because Mus81-Mms4 and MUS81-EME1 have been shown to be involved in the repair of stalled replication forks and cleave 3′ flap and fork-like substrates in vitro, we wished to determine the role of MUS81 in the removal of DPCs.

This study showed that the cleavage of a 3′ flap or a nicked duplex by Mus81-Mms4 and MUS81-EME1 is not impaired by the fluorescein positioned at the 3′ end of the cleaved strand to imitate a remaining active site tyrosyl moiety of TOP1 attached to DNA. Moreover, streptavidin attached to the 3′ end of the cleaved strand also does not prevent MUS81 from cleaving the tested substrates, which contrasts with the substrate containing TOP1 in its native state. Since the structure of TOP1 covalently bound to a short DNA duplex shows the formation of a clamp around DNA with the TOP1-DNA linkage buried inside the protein [52] in contrast to streptavidin that does not bind DNA, our results indicate that the MUS81 nuclease activity is not compromised by the presence of a large protein as long as the cleavage site for MUS81 is accessible. This is further supported by the ability of MUS81 to cleave proteolytically processed TOP1ccs. Similarly, we show that also TDP1 cannot efficiently cleave TOP1 in its native state, in agreement with previously reported data [56–58]. The requirement for the proteolytical processing of DPCs has also been indicated by the fact that the inhibition of the proteasome results in a less-efficient repair of DPCs [59, 60]. CPT-induced DSBs in cells also depend on the proteasomal activity and polyubiquitination of TOP1 [61, 62]. On the other hand, other studies have demonstrated that CPT-dependent DSBs do not depend on proteasomal activity [63, 64]. This might, however, reflect the identification of other proteases involved in processing DPCs. The budding yeast Wss1, a DNA-dependent protease, was shown to play a role in the repair of Top1cc through the proteolysis of Top1 [25]. Cells lacking *WSS1* and *TDP1* present a hypersensitivity to CPT that is suppressed by the disruption of the *TOP1* gene, suggesting that Wss1 acts in a Tdp1-independent repair pathway of Top1-mediated DNA damage [25]. In higher eukaryotes, the metalloprotease SPARTAN, which bears similarities with Wss1 [28], has been proposed to be essential for the DPC repair during replication [18, 29, 30, 65]. Recently, another protease, Ddi1, has been found in yeast to be also involved in the degradation of Top1cc and DPCs in general [66], suggesting a spectrum of proteases required for DPC repair and reflecting the variability of protein-DNA cross-links.

Another possible pathway for making the cleavage site accessible for nucleases could require unfolding or dislodgement of TOP1 from DNA, which is supported by the ability of MUS81 complexes to cleave DNA linked to streptavidin. For example, a helicase or binding proteins may partially displace TOP1, making it more accessible for cleavage. Indeed, we have previously described the ability of the budding yeast Srs2 and Rad54 translocases to directly interact and stimulate Mus81-Mms4 nuclease activity [67, 68]. Similarly, human RecQ5 helicase binds and enhances the enzymatic activity MUS81-EME1 through their physical interaction [69]. In addition, the post-translational modification of TOP1 could lead to a conformational change resulting in the weakening of DNA binding, as has been reported for Rad52 and other proteins [70–72]. Accordingly, the SUMOylation induces TOP2 remodelling and makes the phosphotyrosyl-DNA bond accessible for Tyrosyl-DNA phosphodiesterase 2 (TDP2) hydrolysis [73, 74] and parylation is required for TDP1-dependent repair of TOP1ccs [58].

**Fig. 6:**
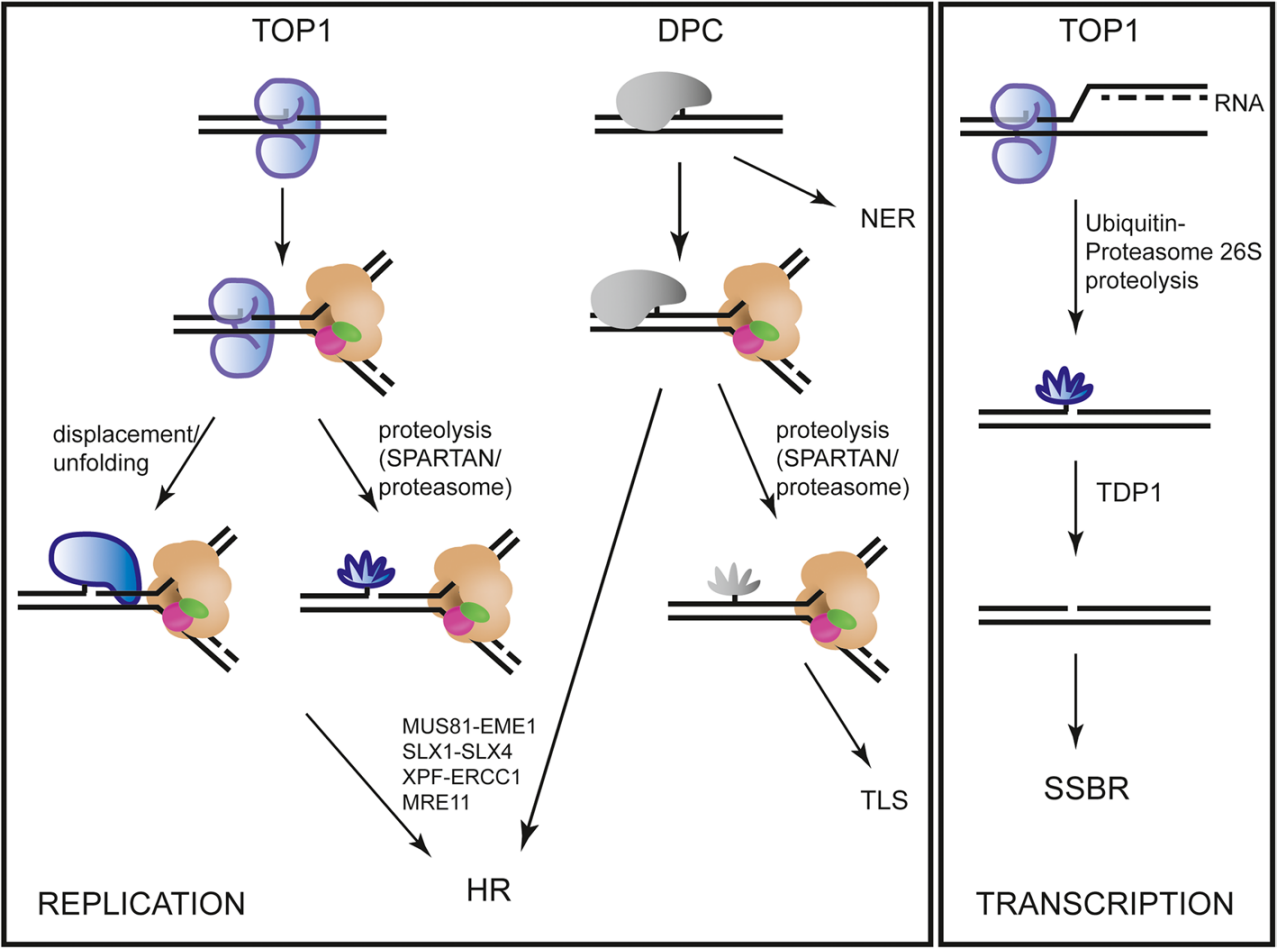
A possible model for TOP1cc and DPC repair. TOP1ccs (blue) and other DPCs (grey) represent a block for the replication CMG complex (beige). To remove a TOP1cc, it has to be either degraded by the proteasome or proteases (i.e. SPARTAN) or alternatively displaced/unfolded from DNA. Next, the processed TOP1 can be cleaved by a nuclease, such as MUS81-EME1, SLX1-SLX4, XPF-ERCC1 or MRE11, followed by homologous recombination (HR). DPCs that NER does not remove can also undergo proteolysis by a protease or proteasome, followed by translesion synthesis (TLS). Alternatively, DPCs can be removed by the action of the nucleases and repaired by HR. When a TOP1cc blocks transcription, TOP1 is degraded in a ubiquitin/proteasome 26S-dependent manner. TDP1 then removes the remaining TOP1 peptide and the break is repaired by a single-strand break repair pathway (SSBR)

The ability of MUS81 to cleave a DPC-mimicking substrate in nuclear extracts indicates that MUS81 could possess the same activity in vivo, contrary to what was proposed before [75]. Their conclusion was based on the fact that no accumulation of TOP1ccs after the treatment of MUS81-depleted cells with CPT was observed, in contrast to the results observed in TDP1-depleted cells [75]. However, the anti-TOP1 antibodies may not detect proteolytically processed TOP1ccs complexes and that higher number of trapped TOP1 could have been detected in MUS81 TDP1 double mutant. Nevertheless, another explanation for this discrepancy might reflect differences in a cell line, cell-cycle phase, DNA structure or metabolic process. The role of MUS81 in processing DPCs is also supported by induced CPT hypersensitivity of MUS81-KO cells upon depletion of TDP1, indicating that MUS81 and TDP1 play parallel roles in the repair of CPT-induced damage. In agreement, MUS81 also appeared to be crucial for DPC repair in plants, where the mus81-1 mutant was highly sensitive to CPT [18]. The role of MUS81 and TDP1 in separate pathways is also supported by the observed proliferation defect in unstressed MUS81- and TDP1-deficient cells. These data are in agreement with the detected reduction in plant size of the tdp1-4 mus81-1 double mutant in *Arabidopsis thaliana* [18]. Based on the available data, we propose a model where TDP1 primarily acts on transcription-associated TOP1ccs and result in the formation of single-strand breaks (SSBs) (Fig. 6). On the other hand, MUS81 generates DSBs at stalled replication forks in response to CPT or DNA-crosslinking agents [45, 75, 76] as well as reversed forks [77]. Alternatively, direct cleavage of DPCs/TOPcc by MUS81 leads to the generation of SSB that can be converted to DSB by replication run-off [15]. However, the formation of single-strand gaps might also represent a substrate for post-replicative repair and representing a hallmark for improving cancer therapy [78]. Finally, Zhang and colleagues suggested TOP1-induced DSBs formation in non-dividing cells by cleavage of R-loops [79]. Indeed, MUS81 could also preferentially act on unrepaired DPCs at the G2/M phase when it is activated by phosphorylation [80]. However, DSB formation would require cooperation with other nucleases, most likely components of the SMX nuclease complex containing SLX1-SLX4/XPF-ERCC1/MUS81-EME1 [81].

Given the broader spectrum of MUS81 substrates, it may also act on other types of DNA-protein cross-links (DPCs), like those that arise upon exposure to agents such as ionising radiation, UV light, metals and reactive aldehydes, including formaldehyde [82]. This might perhaps explain our observation, that in contrast to TDP1, the sensitivity of MUS81-KO or TDP1/MUS81 double mutant to CPT is only partially suppressed by TOP1 depletion, most likely reflecting topoisomerase-independent toxicity of CPT [83, 84]. Homologous recombination (HR) and nucleotide excision repair (NER) have also been identified to be required to repair DPCs in bacteria. Although NER excises the entire DPC but cannot act on large DPCs, HR excises DPCs independent of their size [60, 85]. The function of MUS81 in the resolution of recombination intermediates, together with the required proteolytical processing, suggests its more general role in the repair of various DPCs.

However, co-depleted cells can still proliferate, indicating that other repair pathways exist to deal with DPCs. In budding yeast, it has been shown that Mus81-Mms4, Rad1-Rad10 or Mre11 take part in the repair of Top1ccs in addition to the Tdp1 pathway. The *RAD1*-*TDP1* double mutant showed hypersensitivity to CPT [33], and the *RAD1-TDP1*-*MUS81* triple mutant presented even higher sensitivity [32]. Moreover, assays with budding yeast indicate that also Rad52 is essential for Top1ccs repair [22, 33], reflecting the importance of homologous recombination. Studies using a suicide Top1 mutant indicate that *RAD52* and *TDP1* are epistatic and that there are other Rad52-dependent pathways in addition to the Tdp1 pathway [22]. In addition, an *MRE11* mutant strain appeared to be more sensitive to CPT than the *RAD52* strain, and the sensitivity of this *MRE11* strain is abolished when *TOP1* is disrupted [32]. Indeed, a protein block on DNA was shown to stimulate endonuclease cleavage by the yeast Mre11 complex and could represent a means through which MRE11 plays a role not only in the removal of Spo11 linked to DNA during meiosis [86] but also in the removal of other DPCs. Additionally, it has been shown that TDP2 not only removes TOP2 DPCs but can also process TOP1cc in the absence of TDP1 [87–89].

## Conclusions

Our data show the ability of MUS81 complexes to cleave proteolytically processed TOP1ccs and model DPCs as efficiently as various DNA junctions and point to their direct role not only later in the processing of recombination intermediates but also early in cross-link repair. Persistent TOP1ccs are relevant for the success of chemotherapy because the generation of DSBs is believed to underline the anti-cancer properties of CPT derivatives. However, it may as well lead to the formation of single-strand gaps that represent a cancer hallmark considered for improving cancer therapy outcomes and overcoming resistance [78]. In addition, another topoisomerase poison (irinotecan) combined with a proteasome inhibitor showed higher tumour growth inhibition in mice [90]. Proteasome inhibitors have been found to improve the effect of CPT against colorectal cancer [91] or pancreatic cell lines [92]. CPT derivatives are also being studied for clinical use in combination with DNA damage response inhibitors such as PARP1i, ATRi and CHK1i, or immunotherapy [93]. Therefore, these data suggest that targeting MUS81 and other repair pathways dealing with TOP1ccs or other DPCs may represent a new therapeutic and more specific strategy.

## Methods

### Preparation of unmodified or streptavidin-bound DNA substrates

Synthetic oligonucleotides were purchased from Eurofins Genomics. The sequences and structures are listed in Additional file 7: Table S1 and Additional file 1: Fig. S1, respectively. The fluorescein label at the particular oligonucleotides is indicated by an asterisk. All substrates were prepared according to Marini et al. [94]. Briefly, equimolar amounts of the corresponding oligonucleotides were mixed in hybridising buffer (50 mM Tris, 100 mM NaCl and 10 mM MgCl_2_), heated to 75 °C for 3 min and cooled slowly to room temperature for annealing. The substrates were then purified by HPLC using a 1-mL Mono Q column (GE Healthcare Life Sciences) and a 20-mL gradient in 10 mM Tris buffer containing up to 1 M NaCl. The purity was checked on the native PAGE. The corresponding fractions were then concentrated on a Vivaspin Concentrator 5000 MWCO and washed with buffer W (25 mM Tris and 3 mM MgCl_2_). The concentrations were determined using the absorbance at 260 nm and the corresponding molar extinction coefficients. Streptavidin was conjugated to biotin-labelled substrates by incubation for 1 h at room temperature with an equimolar amount of streptavidin.

### Preparation of native and trypsinised TOP1 suicide DNA substrates

Synthetic oligonucleotides were purchased from Eurofins Genomics. The oligonucleotide oligo 7 (Additional file 3: Fig. S3B, Additional file 7: Table S1), labelled with fluorescein at the 5′ end, contains a preferred TOP1 recognition sequence that allows the cleavage of and covalent binding to TOP1 [95]. After DNA cleavage by TOP1, the last three 3′ end nucleotides are released (to yield oligo 7^−^). Oligonucleotides 8, 9 and 12 were first phosphorylated at the 5′ end to avoid any possible ligation due to TOP1 activity (Additional file 4: Supplementary methods). A nicked DNA duplex, in which the full-length native TOP1 is bound to the 3′ end of the break (Additional file 1: Fig. S1D), was prepared by the hybridisation of oligos 7 (75 pmol), 9 (150 pmol) and 8 (200 pmol) in TOP buffer (5 mM MgCl_2_ and 10 mM Tris, pH 7.5) in a total volume of 75 µL. The mixture was heated to 75 °C and cooled down slowly to RT. Then, 10 pmol of this substrate was incubated with 17 pmol of TOP1 in TOP buffer in a total volume of 600 µL for 2 h at 37 °C. The substrate was then maintained at 4 °C and remained stable for approximately 2 weeks.

Trypsinised substrates to which a protease-resistant TOP1-derived peptide remains covalently attached were prepared by digestion with trypsin (Additional file 1: Fig. S1E). Based on the trypsin-cleavage pattern and TOP1 sequence, we estimate that a 7-amino-acid peptide (from Leu721 to Arg727) remains attached to DNA. The trypsinised nicked duplex was prepared from the native TOP1-nicked duplex described above. The native substrate (180 µL) was precipitated with NaCl/ethanol, and 60 µL of activated trypsin (1 µg/µL in 10 mM Tris, pH 7.8) was then added. The mixture was incubated at 37 °C for 45 min. TOP buffer was added to obtain a final reaction volume of 100 µL, and the sample was heated to 75 °C for 5 min. An additional 3 pmol of oligo 9 and 5 pmol of oligo 8 were added, and the sample was allowed to cool down. Aliquots were maintained at −20 °C. The preparation of the 3′ flap and Y-form with trypsinised TOP1 bound to the 3′ end of the single-stranded part of the substrates (Additional file 1: Fig. S1E) is schematically explained in Additional file 3: Fig. S3A and in Additional file 4: Supplementary methods.

### Expression and purification of TDP1

The TDP1 construct was transformed into BL21 competent cells. The cells were grown in 2 L of 2 × TY media containing 50 µg/mL ampicillin to an A_600_ of 0.9 before protein expression was induced with 1 mM of isopropyl-1-thio-β-D-galactopyranoside. The induction proceeded for 2 h at 37 °C, and the cells were then cooled down for 15 min on ice and harvested by centrifugation. The pellets were flash-frozen in liquid nitrogen and stored at −80 °C.

The purification was performed based on the protocol described previously [96]. The pellet was resuspended in 160 mL of binding buffer (0.5 M NaCl, 5 mM imidazole, 20 mM Tris pH 7.9, 1 mM PMSF and 1 mM DTT) and sonicated. The crude lysate was clarified by centrifugation, and the supernatant was filtered through a 45-μm filter and loaded onto a 2-mL nickel column (Ni–NTA Superflow, Qiagen) previously equilibrated with binding buffer. The column was washed with 20 mL of binding buffer and then with 40 mL of binding buffer containing 60 mM imidazole. The protein was eluted with 20 mL of elution buffer (0.5 M NaCl, 150 mM imidazole and 20 mM Tris, pH 7.9). The eluate was collected in 0.5-mL fractions and stored in 50% glycerol at −20 °C.

### Purification of TOP1

TOP1 was expressed in the yeast *S. cerevisiae* RS190, which lacks the endogenous TOP1 gene and was purified by column chromatography using a heparin sepharose and phenyl sepharose matrix, as described previously [97].

### Purification of MUS81 complexes

The full-length MUS81-EME1 and MUS81-EME2 expression constructs were a kind gift from Stephen West (Cancer Research, UK). The truncated version of MUS81-EME1 used in Additional file 2: Fig. S2A was expressed using MUS81(246-551)-EME1(178-570) expression plasmid, a kind gift of Yunje Cho (Postech, South Corea). Site-directed mutagenesis was used to generate MUS81-nuclease deficient mutant (D338A, D339A) using primers TGCTGCTGCAAAGGGCAGCCAGTCGCTTGCGC and GCGCAAGCGACTGGCTGCCCTTTGCAGCAGCA. The expression was performed as described previously [38], and the purification was performed as published previously [68]. Briefly, the bacterial cells were harvested and resuspended in lysis buffer (50 mM Tris-HCl, pH 7.5, 10% sucrose, 150 mM KCl, 10 mM EDTA, 1 mM DTT, 0.01% Nonidet® P40 (substitute) and protease inhibitors) and sonicated. The lysate was clarified by centrifugation and loaded sequentially into Q-sepharose and SP-sepharose columns (GE Healthcare). The protein was eluted from the SP column with a 150 to 850 mM KCl gradient in buffer K (20 mM K_2_HPO_4_, 10% glycerol, 0.5 mM EDTA, 1 mM DTT and 0.01% Nonidet® P40 (substitute)). The protein-containing fractions were bound to His-Select nickel affinity gel (Sigma-Aldrich) and eluted with increasing concentrations of imidazole (50, 150, 300, 500 mM and 1 M) in buffer K with 150 mM KCl. The fractions with protein were pooled, and their conductivity was adjusted before loading onto a heparin column. The protein complexes were eluted with a gradient from 250 to 900 mM KCl in buffer K, pooled, concentrated and stored in aliquots at −80 °C. The expression and purification of the *S. cerevisiae* Mus81-Mms4 complex were performed as described previously [68]. Briefly, the bacterial cells were resuspended in lysis buffer (50 mM Tris-HCl, pH 7.5, 10% sucrose, 150 mM KCl, 10 mM EDTA, 1 mM DTT, 0.01% Nonidet® P40 (substitute) and protease inhibitors) and sonicated. The crude lysate was clarified by centrifugation. The supernatant was loaded sequentially into Q-sepharose and SP-sepharose columns, and the protein was eluted from the SP column with a 150 to 1000 mM KCl gradient in buffer K. The fractions containing the protein were pooled and bound to a His-Select nickel affinity gel. The complex was eluted with 50, 150 and 300 mM imidazole in buffer K with 150 mM KCl. The fractions containing the complex were pooled, loaded onto a hydroxyapatite column and subsequently eluted with a 0 to 500 mM KH_2_PO_4_ gradient. Finally, the Mus81-Mms4-containing fractions were pooled, loaded onto a MonoS column and eluted with a 150 to 1000 mM KCl gradient in buffer K. The protein complex was concentrated and stored in aliquots at −80 °C. All final pools were checked on SDS-PAGE (Additional file 8: Fig. S6).

### Nuclear extracts

Human breast cancer CAL51 wild-type (WT) and MUS81 knockout (KO) cell lines were cultured in DMEM + 10% FBS, supplemented with L-Glutamine and Penicillin–Streptomycin until confluency. Cells were washed twice with cold PBS, scraped from the flask surface and resuspended in PBS. After spinning the cells down at 4ºC, 1500 RPM for 3 min, PBS was removed and the cells were resuspended in buffer A (20 mM Tris-HCl pH 7.5, 10 mM NaCl, 3 mM MgCl_2_, protease inhibitors). After the addition of Nonidet® P40 (substitute) to 0.4%, the cells were incubated for 20 min in ice. Following centrifugation at 4ºC and 1500 RPM for 8 min, the supernatant corresponding to the cytosolic fraction was removed. The pellet was resuspended in buffer B (20 mM Tris pH 7.5, 3 mM MgCl_2_, 410 mM NaCl, 10% glycerol, 1% Triton-X, protease inhibitors). Sonication was then performed in Bioruptor XL (Diagenode) set to 15 × [15 s + 35 s rest]. After centrifugation at maximal speed, the supernatant, now the nuclear extract, was recovered, aliquoted and stored at −80 °C. Protein concentration was measured by Bradford assay.

### Nuclease assay

The DNA substrate (3 nM fluorescein-only or streptavidin-linked substrates and 5 nM trypsinised substrates) was incubated at 37 °C with the indicated amounts of MUS81-EME1, MUS81-EME2 or Mus81-Mms4 in buffer ME (50 mM Tris pH 7.5, 100 mM KCl, 100 µg/mL BSA, 1 mM DTT and 5 mM MgCl_2_) for 15 min except for the reactions of Mus81-Mms4 with fluorescein-only or streptavidin-linked substrates, which were incubated at 30 °C in buffer MM (20 mM Tris pH 7.5, 100 mM KCl, 100 µg/mL BSA, 0.2 mM DTT, 10 mM MgCl_2_ and 5% glycerol). The reaction was stopped with 0.23% SDS and 0.5 mg/mL proteinase K or only SDS in the case of the trypsinised substrates. The samples were then incubated for an additional 3 min at the corresponding temperature, and this step was followed by the addition of loading buffer (for native gels: 10 mM Tris-HCl, 60% glycerol and 60 mM EDTA; for denaturing gels: formamide and bromphenol blue). The reaction products were then resolved by electrophoresis on a native polyacrylamide gel in TBE buffer. The trypsinised substrates were resolved on 6 M urea-denaturing PAGE. In the reaction with the native TOP1 nicked duplex, increasing concentrations of MUS81-EME1 were incubated with 8 nM substrate at 37 °C for 15 min. The reaction was stopped with 0.6% SDS and incubated for 4 min; the samples were run on a native PAGE, scanned with a FLA-9000 Starion image scanner (Fujifilm) and analysed with Multi Gauge software (Fujifilm). The scanner was later replaced by Amersham Typhoon RGB (Cytiva). For the assay with nuclear extracts, 20 nM of the streptavidin-linked nicked duplex was incubated with the indicated concentrations of CAL51 WT or MUS81-KO nuclear extracts for 1 h at 37 °C. The reaction was stopped by adding SDS and proteinase K, followed by an incubation of 10 min at 37 °C. Loading buffer was added, and samples were resolved on a 16% 6 M urea denaturing PAGE. A control sample with purified MUS81-EME1 was also included.

### Cleavage assays with TDP1

Increasing amounts of TDP1 were incubated with 8 nM native TOP1 nicked duplex at 30 °C for 15 min in buffer T (10 mM Tris pH 8, 100 mM KCl, 1 mM EDTA and 1 mM DTT). The reaction was stopped by adding SDS to a concentration of 0.6% and incubated for 4 min, and the samples were run on a native PAGE.

Similarly, 5 nM trypsinised substrate was incubated at 30 °C with the indicated concentrations of TDP1 in buffer T for 15 min. The reaction was stopped by 0.2% SDS, and the samples were incubated for an additional 3 min. After adding loading buffer (formamide and bromphenol blue), the reaction products were resolved on 6 M urea-denaturing PAGE, and the gels were scanned with a FLA-9000 Starion image scanner (Fujifilm) and analysed with Multi Gauge software (Fujifilm).

### Cell culture and drug treatment

CAL51 MUS81-KO stable cell line was generated by the CRISPR-Cas9 system. First, gRNA sequence CACCGCTGCAGCGGCACCGAACAT was cloned into pSpCas9(BB)-2A-GFP (PX458). Then, CAL51 cells were transiently transfected with the generated plasmid using Lipofectamine 3000 (Thermo Fisher), followed by a selection of GFP-positive single cells using FACS sorter (BD FACS Aria II). The obtained eight clones were later subjected to Western blot, and clone 2 was selected for further use (Additional file 6: Fig. S5A). CAL51 WT and MUS81-KO cell lines were cultured in DMEM + 10% FBS, supplemented with L-Glutamine and Penicillin-Streptomycin. 3 × 10^5^ cells were reverse transfected with 40 nM siRNA pools from Dharmacon either siControl (siCon, #D-001210-01-20), siTDP1 (#M-016112-01-0005) or siTOP1 (OriGene #SR322073) using DharmaFECT 1 reagent (Genetica #T-2001-02) as recommended by the provider. From the transfected-cell suspension, 5 × 10^3^ cells were seeded in each well of a 96-well plate. Parallelly, cells were transfected for knockdown assessment by Western blot in a 6-well plate. The cells were allowed to attach and grow for 3–4 days, at the end of which, cell proliferation was assessed using WST-1 (Roche #11644807001) assay as per the manufacturer’s protocol.

### Western blot analysis

CAL51 WT and MUS81-KO cells were harvested 3–4 days post-transfection with the corresponding siRNA, washed in PBS and lysed in RIPA buffer (Sigma Aldrich # R0278) supplemented with protease and phosphatase inhibitors (Roche; # 000000011836170001 and # 04906837001). Protein concentration was assessed by Bradford assay, and 20–30 µg protein was resolved on a 12% SDS-PAGE. Following antibodies were used to detect TDP1, MUS81 and TOP1: anti-TDP1 (Abcam #ab4166, 1:1000 solution), anti-TOP1 (Abcam #ab109374, 1:10000 solution), anti-MUS81 (Abcam #ab14387, 1:1000 solution) and anti-Actin antibodies (Abcam #ab8226, 1:5000 solution).

## Supplementary Information

**Additional file 1: Figure S1.** Schematics of the DNA substrates used in this study. Each substrate contains one fluorescently labelled oligonucleotide. The 5’ end of this fluorescent oligonucleotide is marked. The numbers in italics represent the numbers assigned to the oligonucleotides as indicated in Additional file 7: Table S1. Oligo 7^-^ lacks the last three nucleotides after TOP1 cleavage.Substrates labelled with fluorescein at the 3’ position.Standard 3’ flap substrate.Substrates modified with biotinto which streptavidinis attached.Nicked duplex with native TOP1 bound to the 3’ end of the nick.Substrates that have been treated with trypsin to degrade TOP1 to leave only a tiny peptide bound to the DNA.Substrate modified with biotinto which streptavidinis attached. Free ends are modified with three consecutive thio-bonds.

**Additional file 2: Figure S2.** Processing of nicked duplexes by MUS81 complexes.Nuclease activity of MUS81-EME1wild-typeor nuclease-deadon nicked duplex labelled with CY5and fluorescein. Products were resolved in native PAGE, and the gel was scanned for both fluorescent labels and images were overlaid. The gel was artificially coloured by the processing software.Nuclease activity of MUS81-EME1on two nicked duplexes, one with fluorescein at the 3’ end of the nick and the other with fluorescein at 5’end of the same oligonucleotide. The bottom strand was labelled with CY5 in both cases. Products were resolved in denaturing PAGE, and the gel was scanned for both fluorescent labels. Oligos of the indicated lengths and labelled with fluorescein were used as marker.

**Additional file 3: Figure S3.** Preparation of trypsinised substrates.Schematic of the steps required to prepare Y-form and 3’ flap trypsinised substrates, as explained in the Methods section.The sequence of oligo 7 shows the TOP1 cleaving site. The last three nucleotides AGA are cleaved off with TOP1 enzyme linked to the shorter oligonucleotide denoted oligo 7^−^.Three different structures were generated: nicked duplex, Y-form and 3’ flap. The denaturing gel of the trypsinised substrates with two major bands: the faster-migrating band corresponds to the unmodified oligonucleotide, indicating a not fully efficient reaction of TOP1 with DNA, and the slower-migrating bandcorresponds to the oligonucleotide bearing the TOP1 peptide. All of the bands correspond to the fluorescent single-stranded oligonucleotide.

**Additional file 4: Supplementary methods.** Additional information for the preparation of the trypsinised substrate. Phosphorylation of oligonucleotides; Preparation of 3’ flap and Y-form with trypsinised TOP1.

**Additional file 5: Figure S4.** Quantification of MUS81 nuclease activity on 3’flap unmodified or bearing a peptide. Quantification of MUS81-EME1, MUS81-EME2and Mus81-Mms4nuclease activities on a 3’ flap substrate. Comparison of the 3’ flapand the 3’ flap carrying the TOP1 peptide after trypsin treatment.

**Additional file 6: Figure S5.** Additional cell-based experiments data.Verification of MUS81 knockout clones by Western blot. Clone 2 was chosen for further experiments. Actin was used as a loading reference.Sensitivity of CAL51 and CAL51 MUS81-KO cells to the indicated concentrations of CPT, withor without TDP1depletion, measured by WST-1 assay. DMSOwas added to the control cells. For each cell line, the results were normalised to the control cells. The means and standard deviations from three independent experiments are shown. The P valueswere calculated through a multiple unpaired t-test. Individual data values can be found in Additional file 10.

**Additional file 7: Table S1.** Oligonucleotides constituting the substrates used in this work. A list of sequences of oligonucleotidesused for individual synthetic substrates is depicted in Additional file 1: Fig. S1. The end modifications -fluand -biotin are indicated, as well as the thio-bonds. The auxiliary oligonucleotides were used to prepare the trypsinised substrates Y-form and 3’ flap but do not constitute the substrates.

**Additional file 8: Figure S6.** Quality of MUS81 complexes assessed by SDS-PAGE.SDS-PAGE of full-length budding yeast Mus81-Mms4and human MUS81-EME1and MUS81-EME2stained with Coomassie blue and scanned using Typhoon RGB imager.SDS-PAGE of truncated human complex MUS81-EME1wild-typeand nuclease-deadstained with Coomassie® Brilliant Blue R-250 and scanned using Typhoon RGB imager. Protein markerof the indicated molecular weights was included.

**Additional file 9.** Uncropped images of gels and Western blots. Uncropped images of gels and Western blots shown in this paper.

**Additional file 10.** Individual data values. Individual data values used for the graphs present in this paper.

## Data Availability

All data generated or analysed in this study are included in this published article and its supplementary information files. Uncropped gels and blots are provided in Additional file 9. Individual data values for each graph can be found in Additional file 10.

## Abbreviations

DPC: DNA-protein cross-link
CPT: Camptothecin
DSB: Double-strand break
SSB: Single-strand breaks
NER: Nucleotide excision repair
KO: Knockout
PAGE: Polyacrylamide gel electrophoresis
HPLC: High-performance liquid chromatography
PMSF: Phenylmethylsulfonyl fluoride
DTT: Dithiotreitol
BSA: Bovine serum albumin
EDTA: Ethylenediaminetetraacetic acid
FBS: Fetal bovine serum
PBS: Phosphate-buffered saline
TBE: Tris/Borate/EDTA buffer
SDS: Sodium dodecyl sulphate
GFP: Green fluorescent protein
DMEM: Dulbecco’s modified Eagle medium
RIPA: Radioimmunoprecipitation assay buffer
siRNA: Small interfering RNA
oligo: Oligonucleotide
