# Supplementary figures and images for: MUS81 cleaves TOP1-derived lesions and other DNA–protein cross-links
Source: BMC Biol. 2023 May 16;21:110. doi: 10.1186/s12915-023-01614-1 (PMC10189953; doi:10.1186/s12915-023-01614-1)

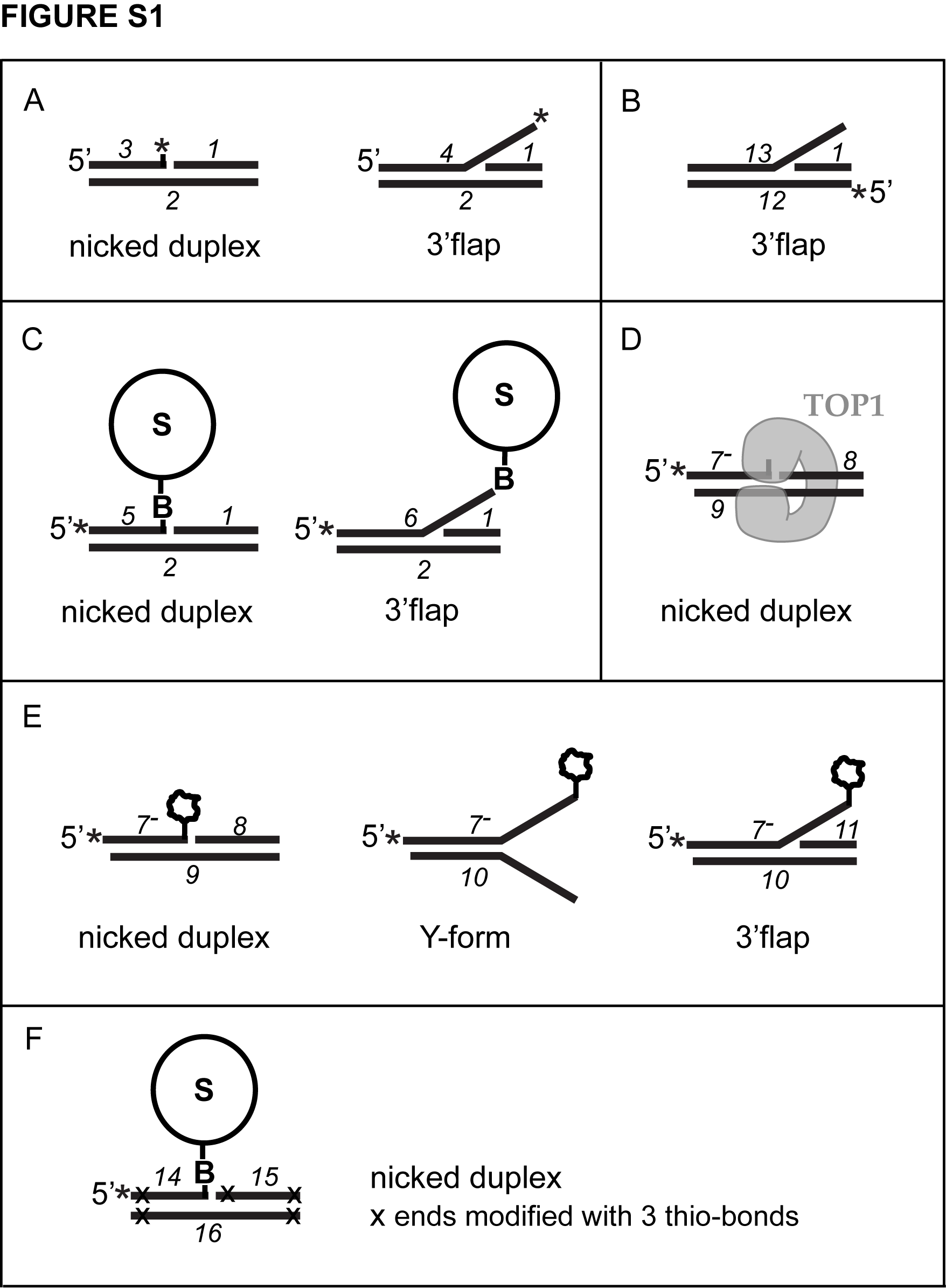

Supplement: Supplementary file 1 — Additional file 1: Figure S1. Schematics of the DNA substrates used in this study. Each substrate contains one fluorescently labelled oligonucleotide. The 5’ end of this fluorescent oligonucleotide is marked. The numbers in italics represent the numbers assigned to the oligonucleotides as indicated in Additional file 7: Table S1. Oligo 7- lacks the last three nucleotides after TOP1 cleavage.Substrates labelled with fluorescein at the 3’ position.Standard 3’ flap substrate.Substrates modified with biotinto which streptavidinis attached.Nicked duplex with native TOP1 bound to the 3’ end of the nick.Substrates that have been treated with trypsin to degrade TOP1 to leave only a tiny peptide bound to the DNA.Substrate modified with biotinto which streptavidinis attached. Free ends are modified with three consecutive thio-bonds. [file 12915_2023_1614_MOESM1_ESM.tif]

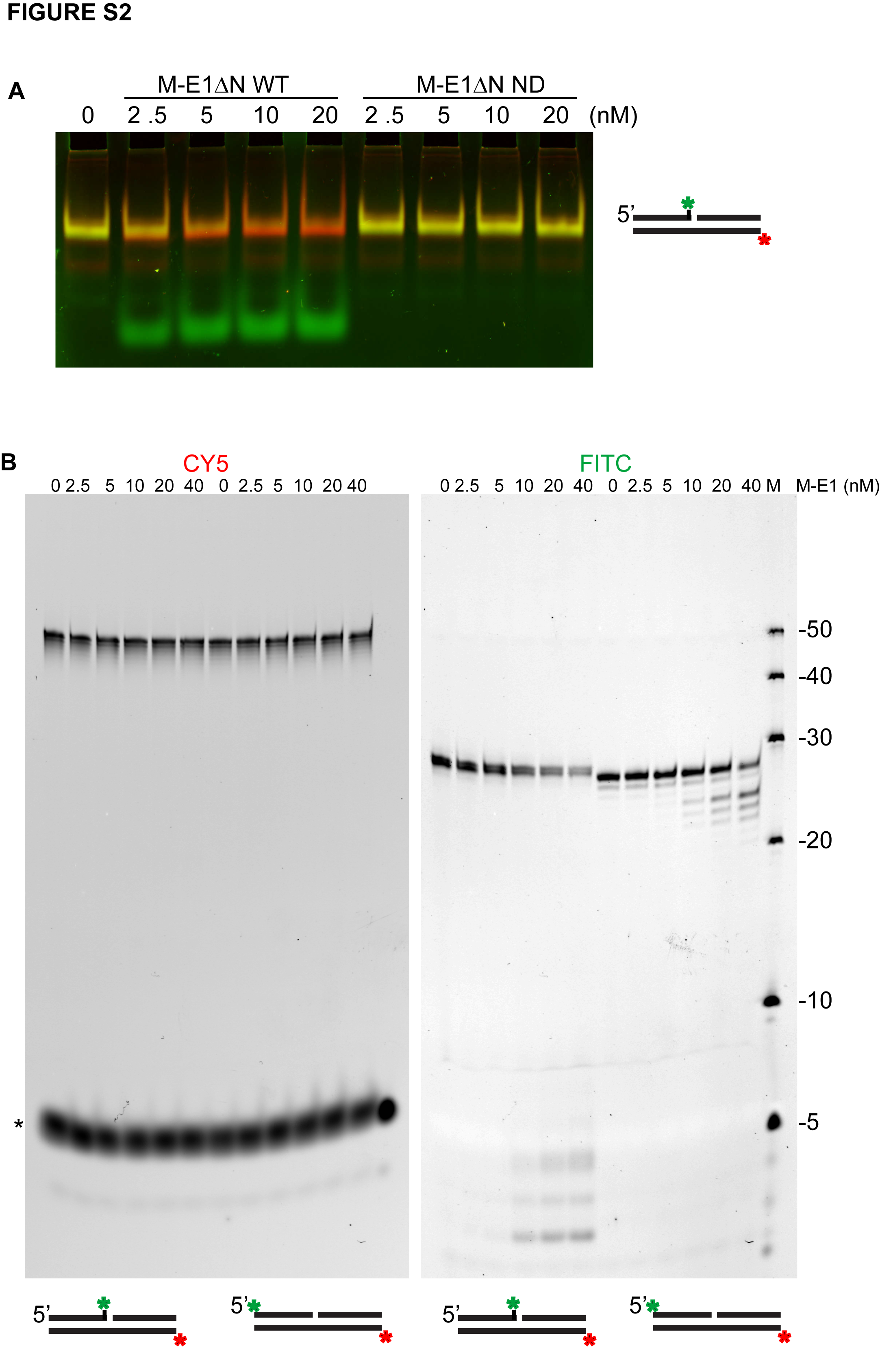

Supplement: Supplementary file 2 — Additional file 2: Figure S2. Processing of nicked duplexes by MUS81 complexes.Nuclease activity of MUS81-EME1wild-typeor nuclease-deadon nicked duplex labelled with CY5and fluorescein. Products were resolved in native PAGE, and the gel was scanned for both fluorescent labels and images were overlaid. The gel was artificially coloured by the processing software.Nuclease activity of MUS81-EME1on two nicked duplexes, one with fluorescein at the 3’ end of the nick and the other with fluorescein at 5’end of the same oligonucleotide. The bottom strand was labelled with CY5 in both cases. Products were resolved in denaturing PAGE, and the gel was scanned for both fluorescent labels. Oligos of the indicated lengths and labelled with fluorescein were used as marker. [file 12915_2023_1614_MOESM2_ESM.tif]

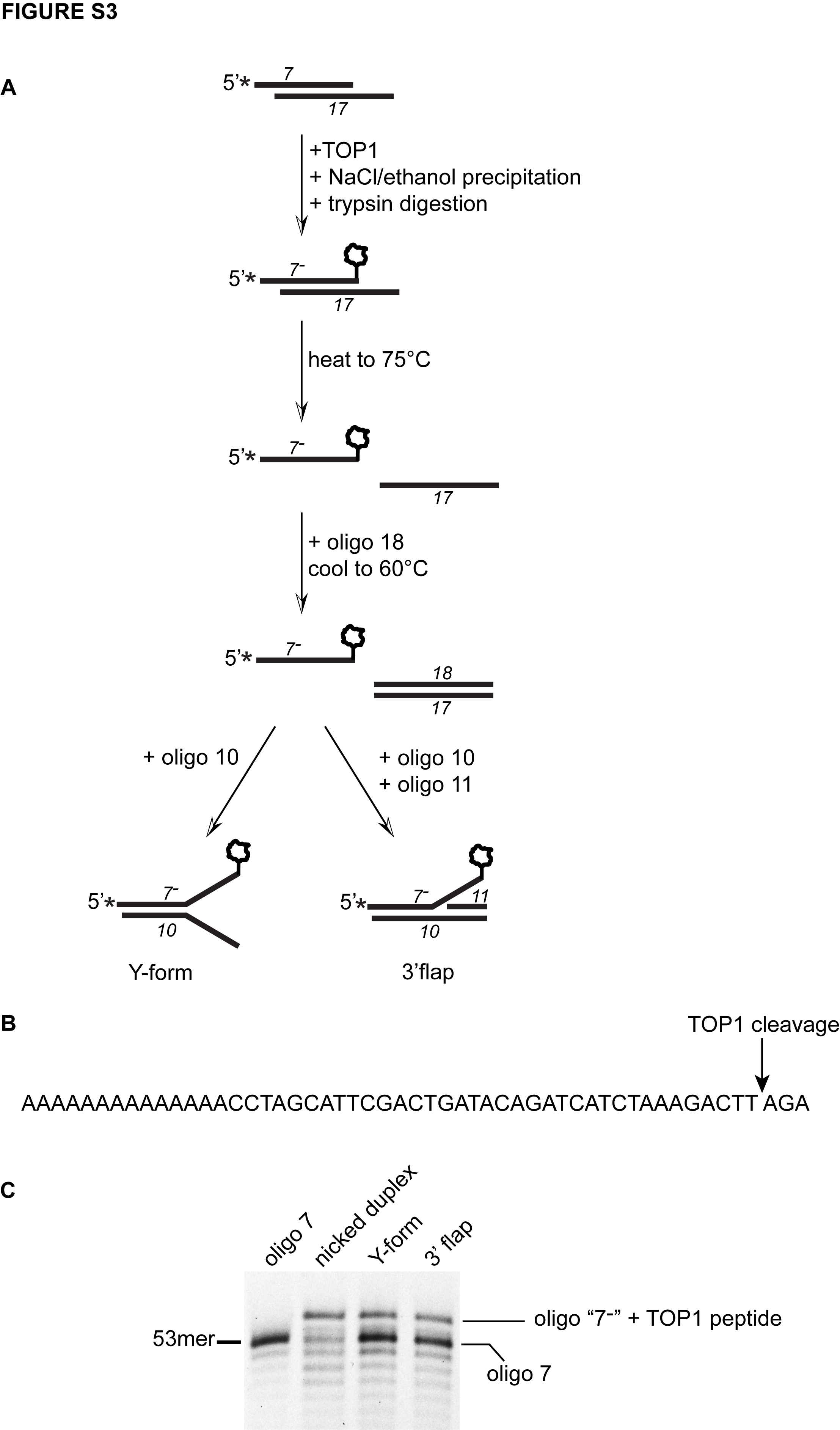

Supplement: Supplementary file 3 — Additional file 3: Figure S3. Preparation of trypsinised substrates.Schematic of the steps required to prepare Y-form and 3’ flap trypsinised substrates, as explained in the Methods section.The sequence of oligo 7 shows the TOP1 cleaving site. The last three nucleotides AGA are cleaved off with TOP1 enzyme linked to the shorter oligonucleotide denoted oligo 7−.Three different structures were generated: nicked duplex, Y-form and 3’ flap. The denaturing gel of the trypsinised substrates with two major bands: the faster-migrating band corresponds to the unmodified oligonucleotide, indicating a not fully efficient reaction of TOP1 with DNA, and the slower-migrating bandcorresponds to the oligonucleotide bearing the TOP1 peptide. All of the bands correspond to the fluorescent single-stranded oligonucleotide. [file 12915_2023_1614_MOESM3_ESM.tif]

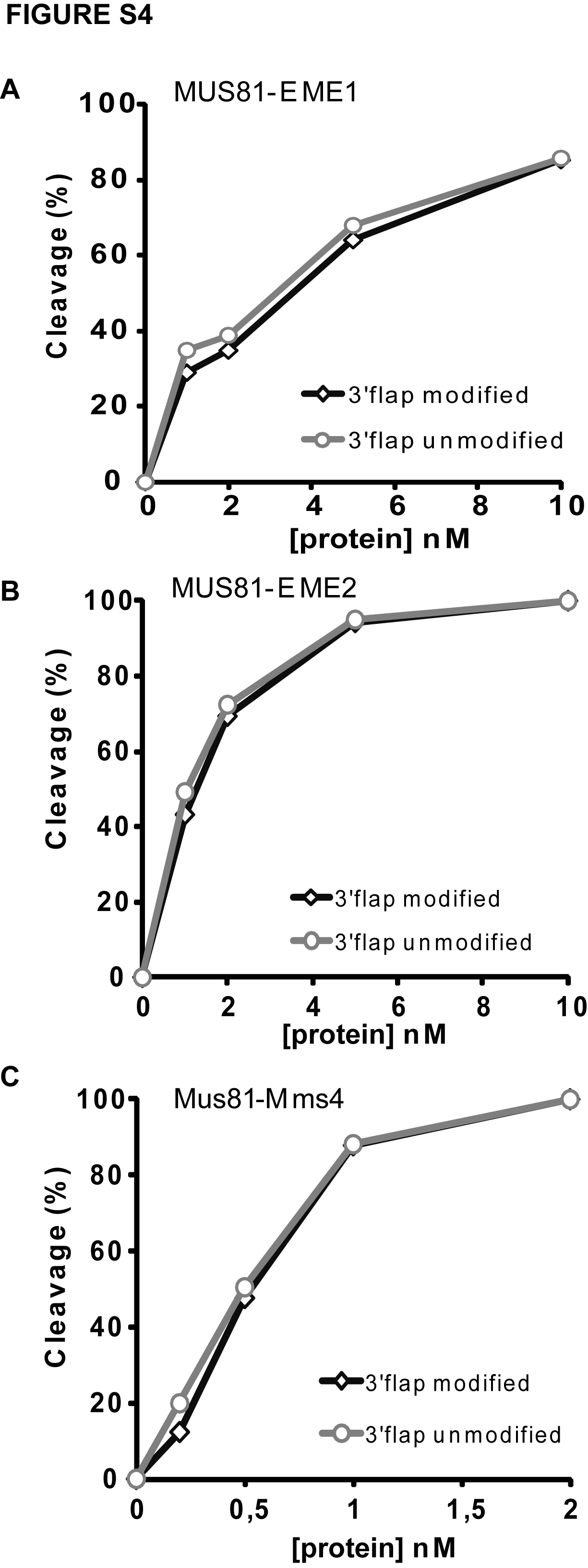

Supplement: Supplementary file 5 — Additional file 5: Figure S4. Quantification of MUS81 nuclease activity on 3’flap unmodified or bearing a peptide. Quantification of MUS81-EME1, MUS81-EME2and Mus81-Mms4nuclease activities on a 3’ flap substrate. Comparison of the 3’ flapand the 3’ flap carrying the TOP1 peptide after trypsin treatment. [file 12915_2023_1614_MOESM5_ESM.tif]

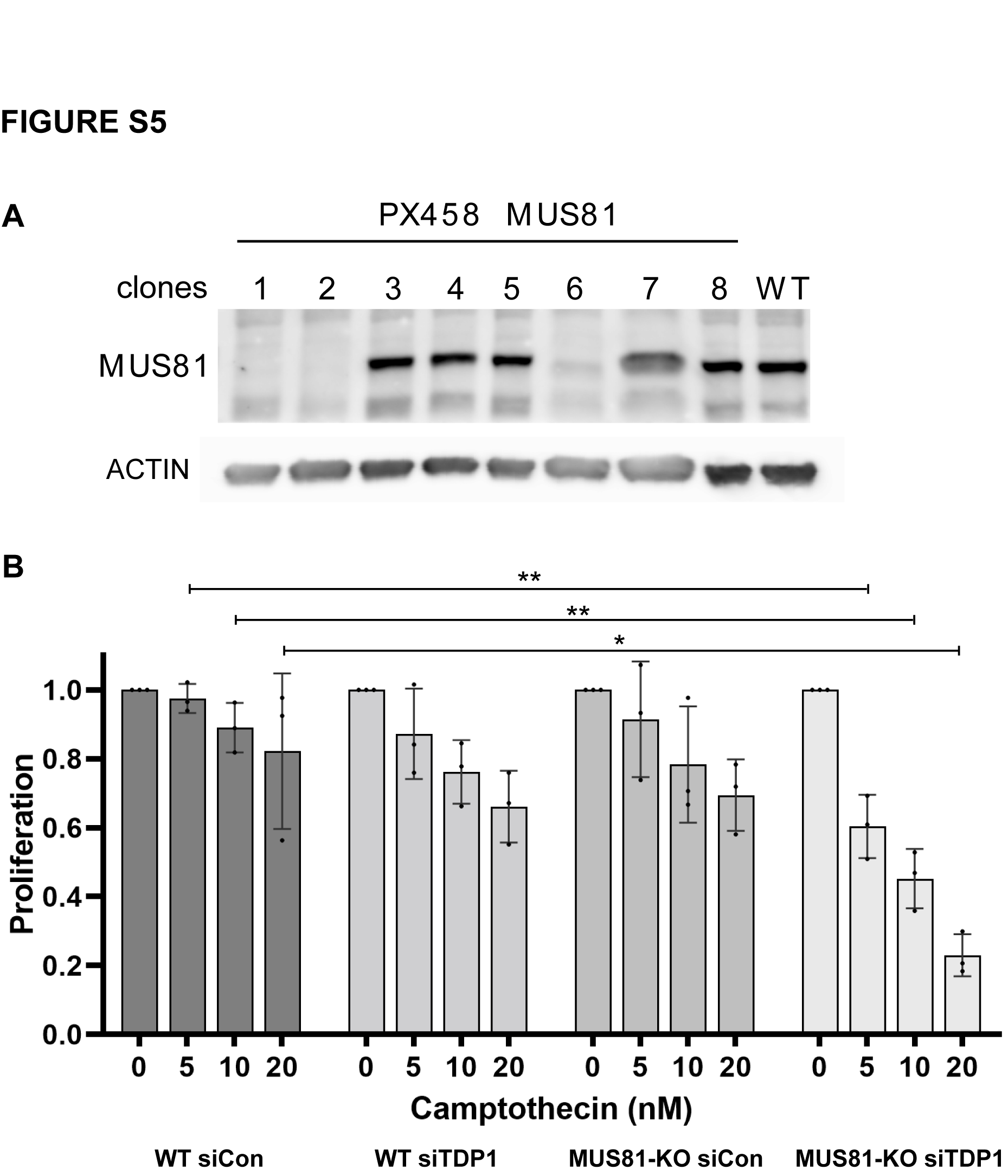

Supplement: Supplementary file 6 — Additional file 6: Figure S5. Additional cell-based experiments data.Verification of MUS81 knockout clones by Western blot. Clone 2 was chosen for further experiments. Actin was used as a loading reference.Sensitivity of CAL51 and CAL51 MUS81-KO cells to the indicated concentrations of CPT, withor without TDP1depletion, measured by WST-1 assay. DMSOwas added to the control cells. For each cell line, the results were normalised to the control cells. The means and standard deviations from three independent experiments are shown. The P valueswere calculated through a multiple unpaired t-test. Individual data values can be found in Additional file 10. [file 12915_2023_1614_MOESM6_ESM.tif]

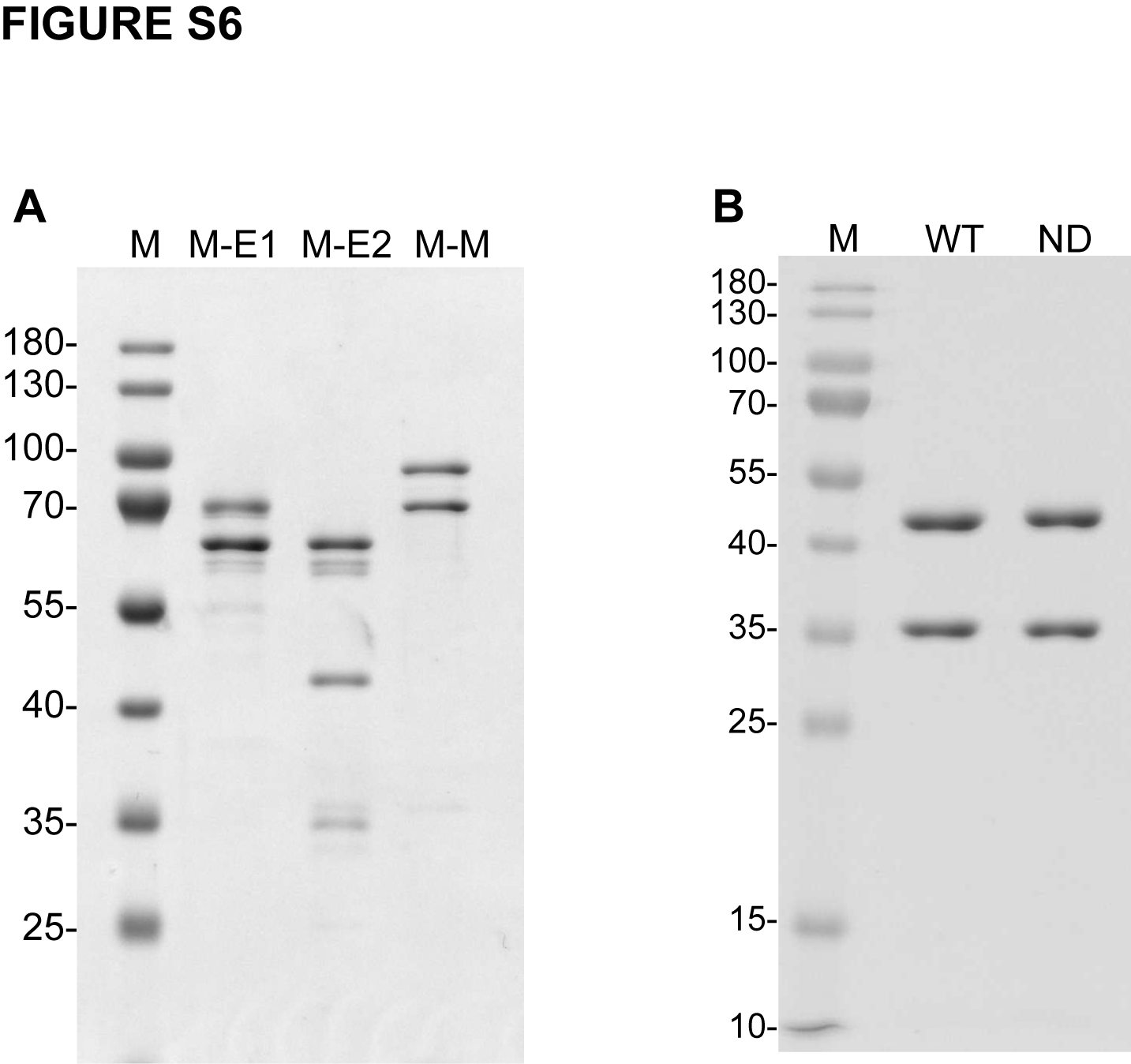

Supplement: Supplementary file 8 — Additional file 8: Figure S6. Quality of MUS81 complexes assessed by SDS-PAGE.SDS-PAGE of full-length budding yeast Mus81-Mms4and human MUS81-EME1and MUS81-EME2stained with Coomassie blue and scanned using Typhoon RGB imager.SDS-PAGE of truncated human complex MUS81-EME1wild-typeand nuclease-deadstained with Coomassie® Brilliant Blue R-250 and scanned using Typhoon RGB imager. Protein markerof the indicated molecular weights was included. [file 12915_2023_1614_MOESM8_ESM.tif]
