# Supplementary material for: MUS81 cleaves TOP1-derived lesions and other DNA–protein cross-links
Source: BMC Biol. 2023 May 16;21:110. doi: 10.1186/s12915-023-01614-1 (PMC10189953; doi:10.1186/s12915-023-01614-1)
Supplement: Supplementary file 4 — Additional file 4: Supplementary methods. Additional information for the preparation of the trypsinised substrate. Phosphorylation of oligonucleotides; Preparation of 3’ flap and Y-form with trypsinised TOP1. [file 12915_2023_1614_MOESM4_ESM.docx]

**SUPPLEMENTARY METHODS**

***Phosphorylation of oligonucleotides***

Individual oligonucleotides (100 pmol) were mixed with 1 mM ATP and 10 units of T4 polynucleotide kinase in reaction buffer (70 mM Tris-HCl, 10 mM MgCl_2_, and 5 mM DTT, pH 7.6) for 30 min at 37°C in a total volume of 50 µL. The DNA was then precipitated by the addition of 5 µL of 3 M NaCl and 140 µL of ice-cold 96% ethanol. The sample was incubated overnight at -20°C and then spun down at the maximum speed for 30 min at 4°C. After carefully removing the supernatant, 500 µL of ice-cold 80% ethanol was added, and the mixture was spun again for 10 min at 4°C. The supernatant was removed, and the sample was allowed to dry.

***Preparation of 3’ flap and Y-form with trypsinised TOP1***

First, we prepared an auxiliary starting native substrate through the hybridisation of oligo 7 (75 pmol) and oligo 17 (150 pmol) in TOP buffer. Then, 30 pmol of this substrate was allowed to react with 4.5 µg of TOP1 using a protocol similar to that described in Material and Methods. The sample was divided into two halves, and after precipitation with NaCl/ethanol, TOP1 was digested with activated trypsin. Both samples were then heated to 75°C to denature the substrate, and oligo 18 was added to the mixtures. Once the samples reached 60°C, oligo 10 (30 pmol) was added to the first tube to form the Y-form substrate, whereas oligo 10 (30 pmol) and oligo 11 (40 pmol) were added to the second tube to create the 3’ flap substrate. Both mixtures were allowed to slowly cool down to room temperature, and small aliquots were maintained at -20°C.
