# Supplementary material for: MUS81 cleaves TOP1-derived lesions and other DNA–protein cross-links
Source: BMC Biol. 2023 May 16;21:110. doi: 10.1186/s12915-023-01614-1 (PMC10189953; doi:10.1186/s12915-023-01614-1)
Supplement: Supplementary file 7 — Additional file 7: Table S1. Oligonucleotides constituting the substrates used in this work. A list of sequences of oligonucleotidesused for individual synthetic substrates is depicted in Additional file 1: Fig. S1. The end modifications -fluand -biotin are indicated, as well as the thio-bonds. The auxiliary oligonucleotides were used to prepare the trypsinised substrates Y-form and 3’ flap but do not constitute the substrates. [file 12915_2023_1614_MOESM7_ESM.docx]

**Table S1**

**Oligonucleotides constituting the substrates used in this work**

| Oligo 1 | AATTCGTGCAGGCATGGTAGCT |
| --- | --- |
| Oligo 2 | AGCTACCATGCCTGCACGAATTAAGCAATTCGTAATCATGGTCATAGCT |
| Oligo 3 | AGCTATGACCATGATTACGAATTGCTT-flu |
| Oligo 4 | AGCTATGACCATGATTACGAATTGCTTGGAATCCTGA-flu |
| Oligo 5 | flu-AGCTATGACCATGATTACGAATTGCTT-biotin |
| Oligo 6 | flu-AGCTATGACCATGATTACGAATTGCTTGGAATCCTGACGAACTGTAG-biotin |
| Oligo 7 | flu-AAAAAAAAAAAAAACCTAGCATTCGACTGATACAGATCATCTAAAGACTTAGA |
| Oligo 8 | AGAAAAATTTTTGATCGTACGCTAG |
| Oligo 9 | CTAGCGTACGATCAAAAATTTTTCTAAGTCTTTAGATGATCTGTATCAGTCGAATG |
| Oligo 10 | CAGTTTATTGCTCAGTGGTACTATCAGTCGAATGCTAGG |
| Oligo 11 | GTACCACTGAGCAATAAACTG |
| Oligo 12 | flu-AGCTACCATGCCTGCACGAATTAAGCAATTCGTAATCATGGTCATAGCT |
| Oligo 13 | AGCTATGACCATGATTACGAATTGCTTGGAATCCTGACGAACTGTAG |
| Oligo 14 | flu-C*A*G*ATCATCTAAAGACTT-biotin |
| Oligo 15 | A*G*A*AAAATTTTTGATCGTACGC*T*A*G |
| Oligo 16 | C*T*A*GCGTACGATCAAAAATTTTTCTAAGTCTTTAGATGAT*C*T*G |
| Auxiliary oligonucleotides | |
| Oligo 17 | AAAAATTTTTCTAAGTCTTTAGATGATCTGTAT |
| Oligo 18 | ATACAGATCATCTAAAGACTTAGAAAAATTTTT |

List of sequences of oligonucleotides (from 5’ to 3’) used for individual synthetic substrates depicted in Additional file 1: Figure S1. The end modifications -flu (for fluorescein) and -biotin are indicated, as well as the thio-bonds (*). The auxiliary oligonucleotides were used to prepare the trypsinised substrates Y-form and 3’ flap but do not constitute the substrates.
