# Supplementary material for: MUS81 cleaves TOP1-derived lesions and other DNA–protein cross-links
Source: BMC Biol. 2023 May 16;21:110. doi: 10.1186/s12915-023-01614-1 (PMC10189953; doi:10.1186/s12915-023-01614-1)

Figure 1

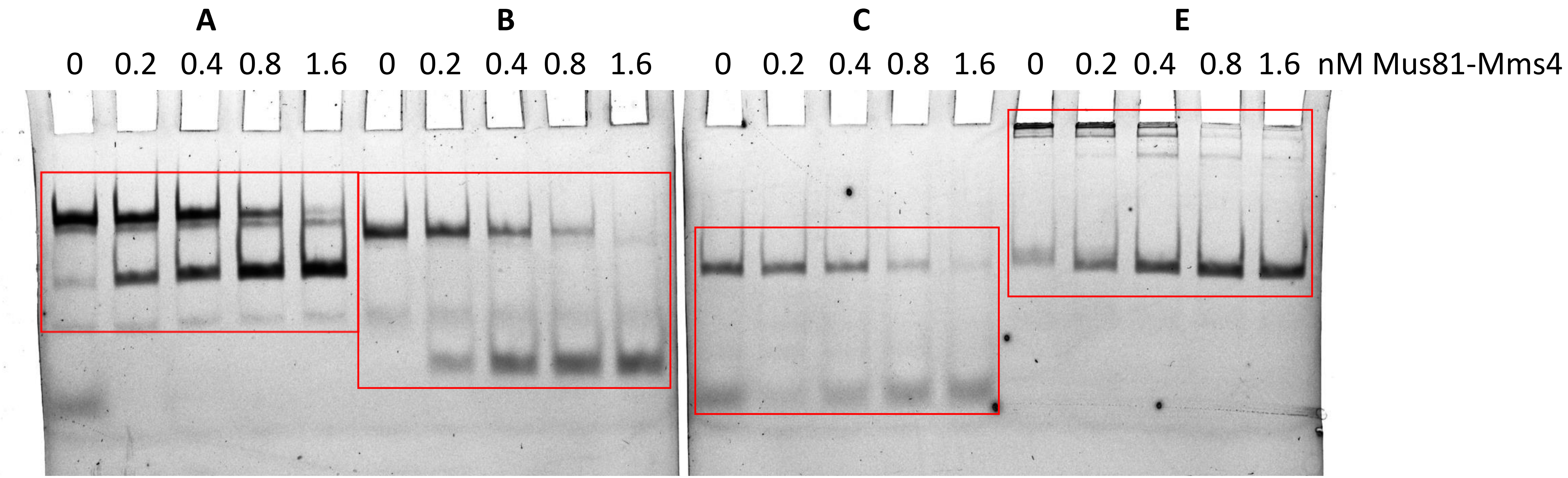

A
B
C
E
0 0.2 0.4 0.8 1.6 0 0.2 0.4 0.8 1.6 0 0.2 0.4 0.8 1.6 0 0.2 0.4 0.8 1.6 nM Mus81-Mms4

Figure 1

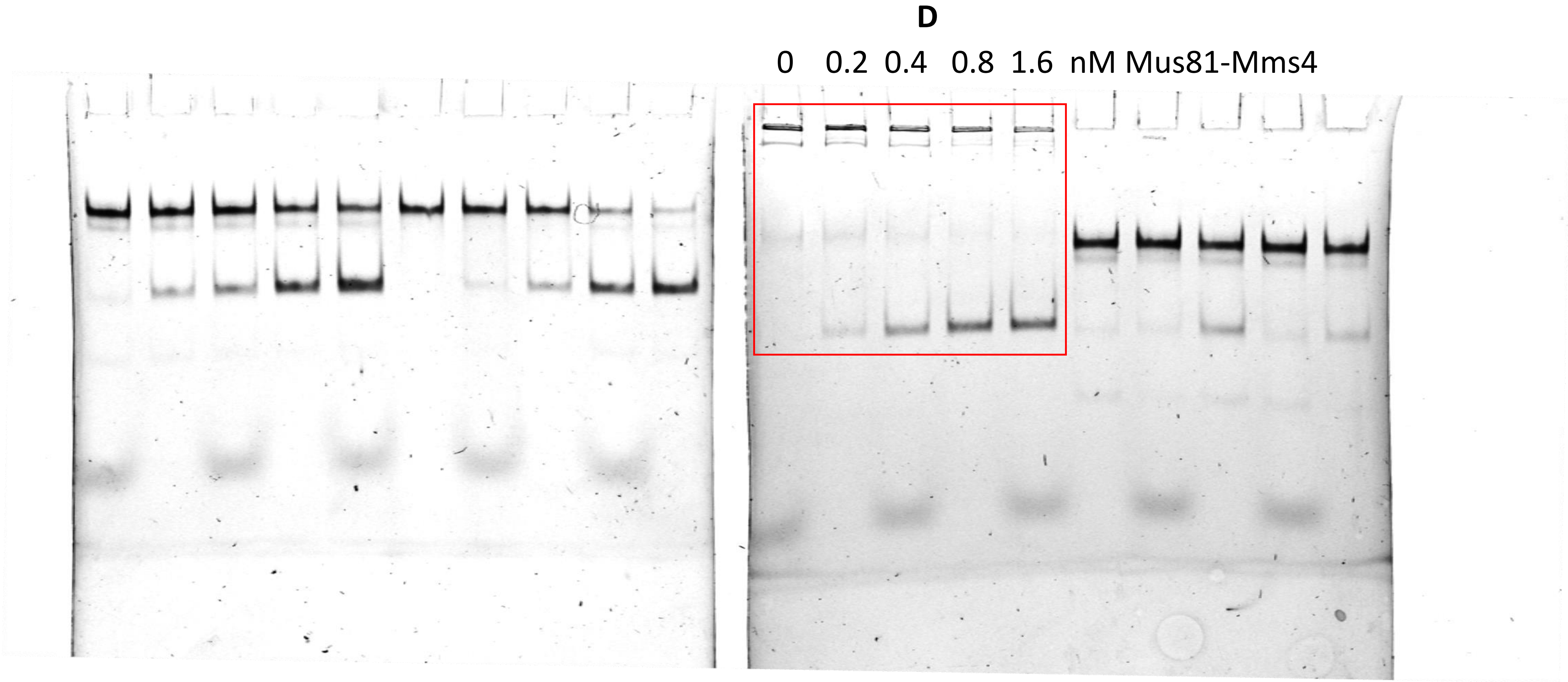

Figure 1

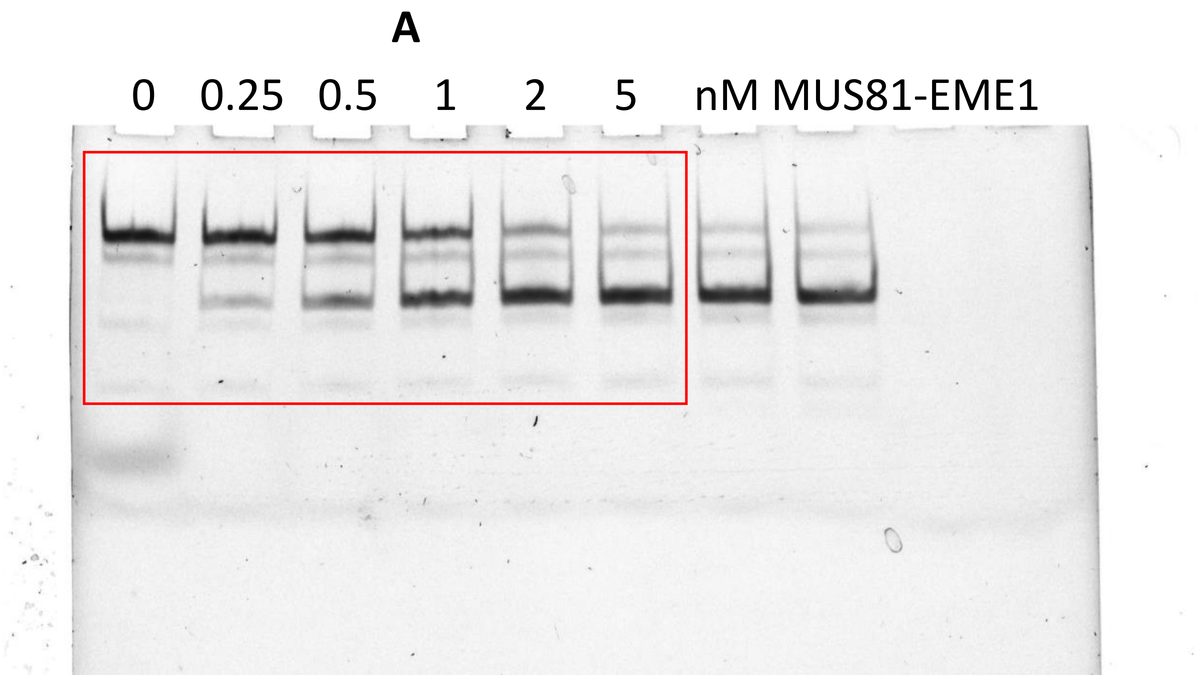

Figure 1

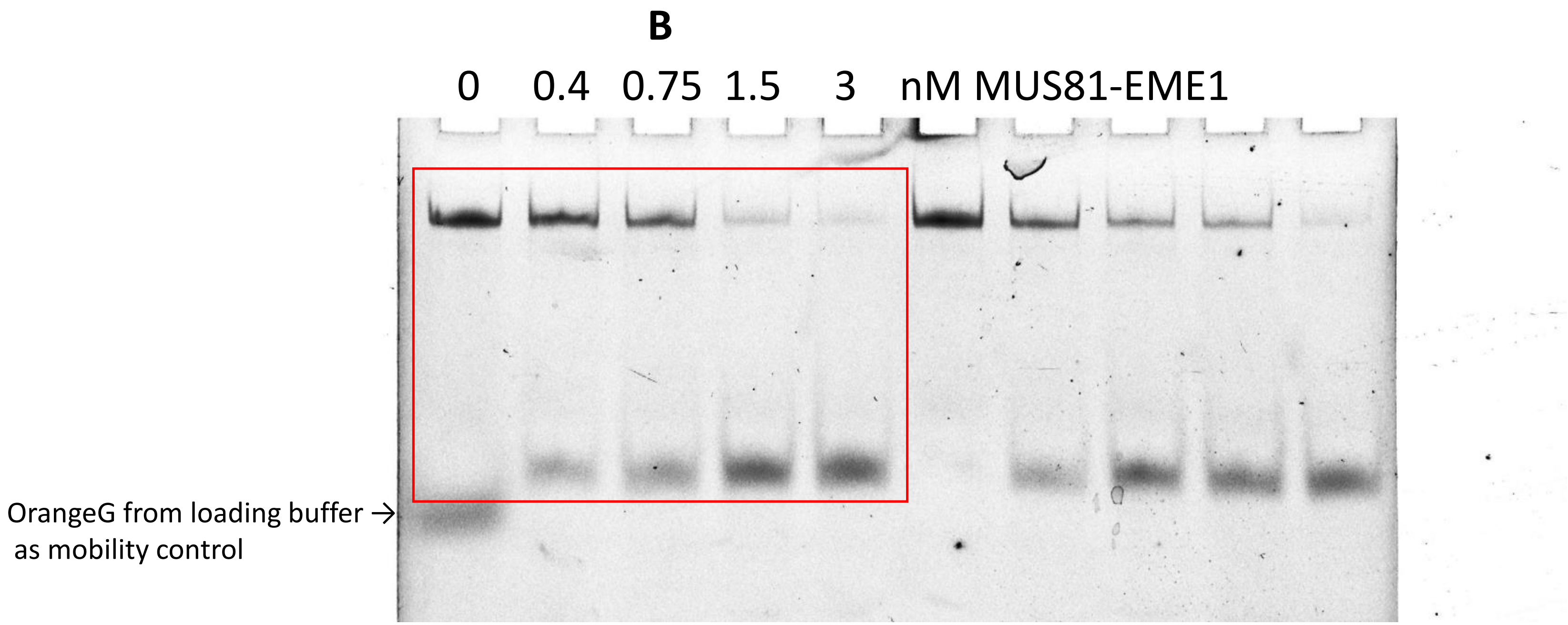
B
0 0.4 0.75 1.5 3 nM MUS81-EME1
OrangeG from loading buffer as mobility control

Figure 1

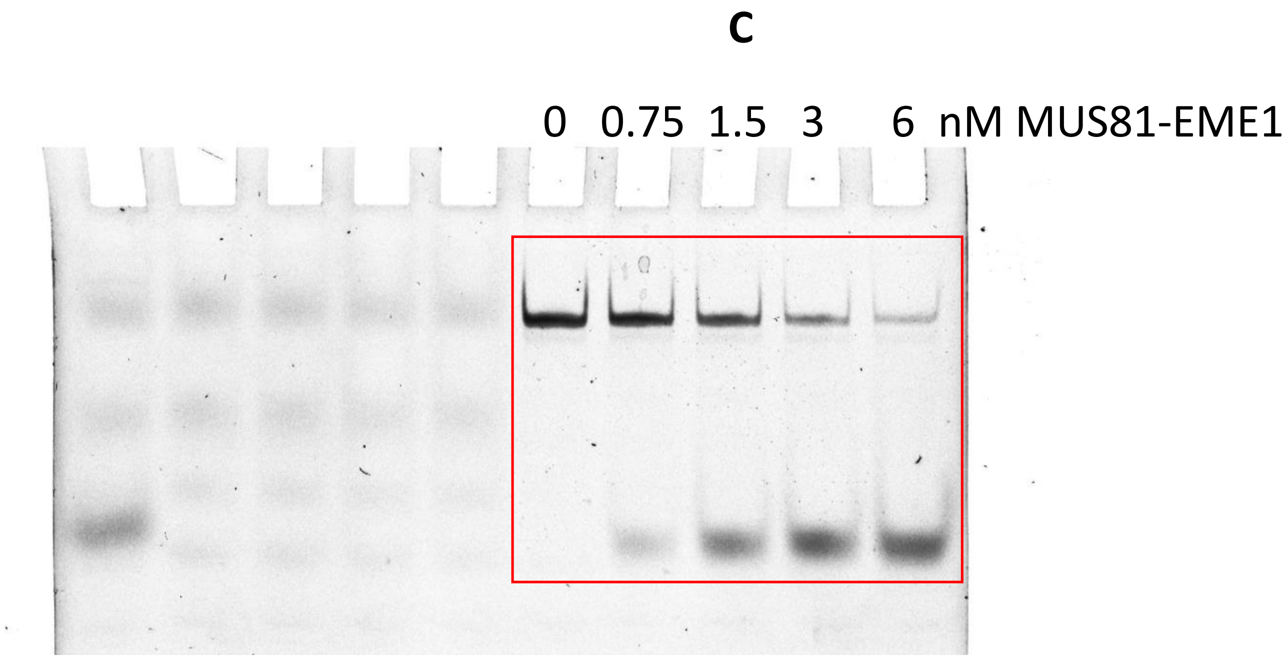

Figure 1

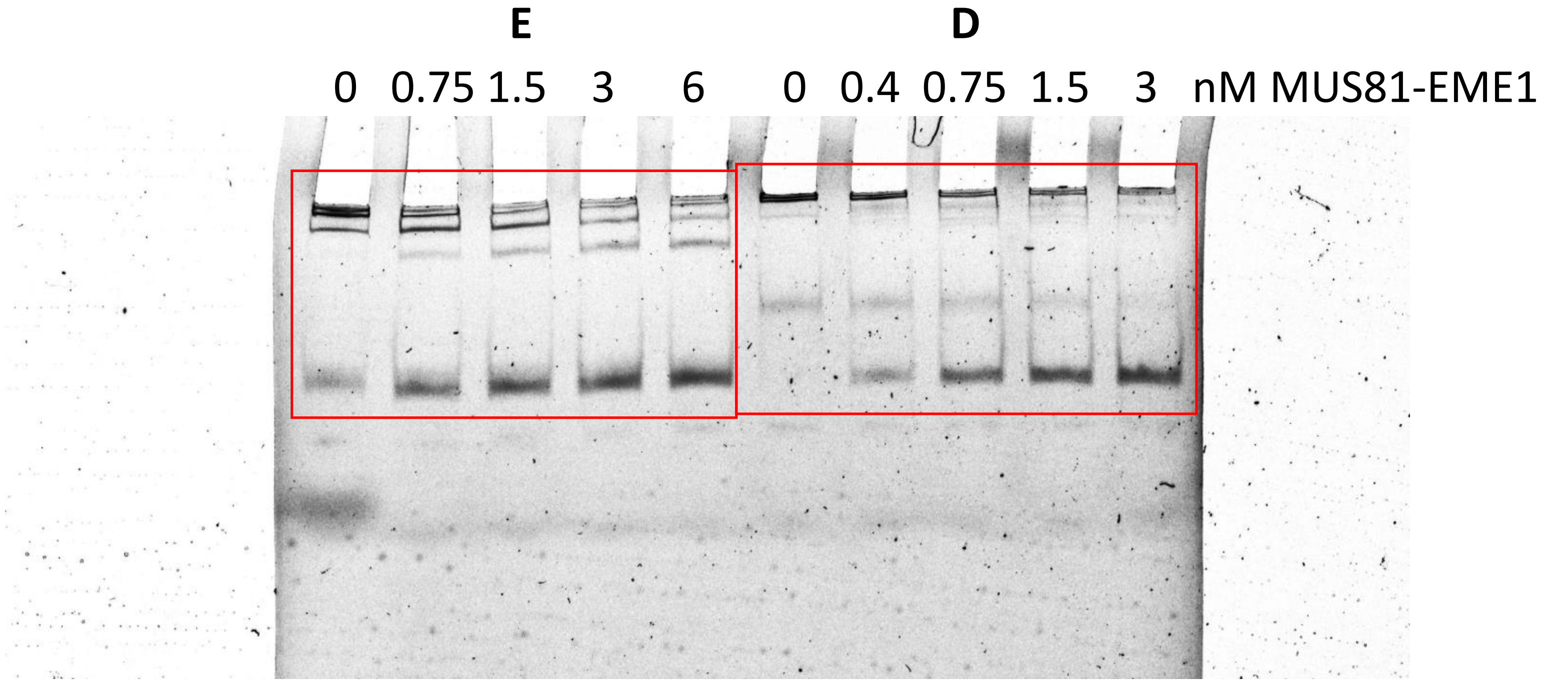

Figure 2A

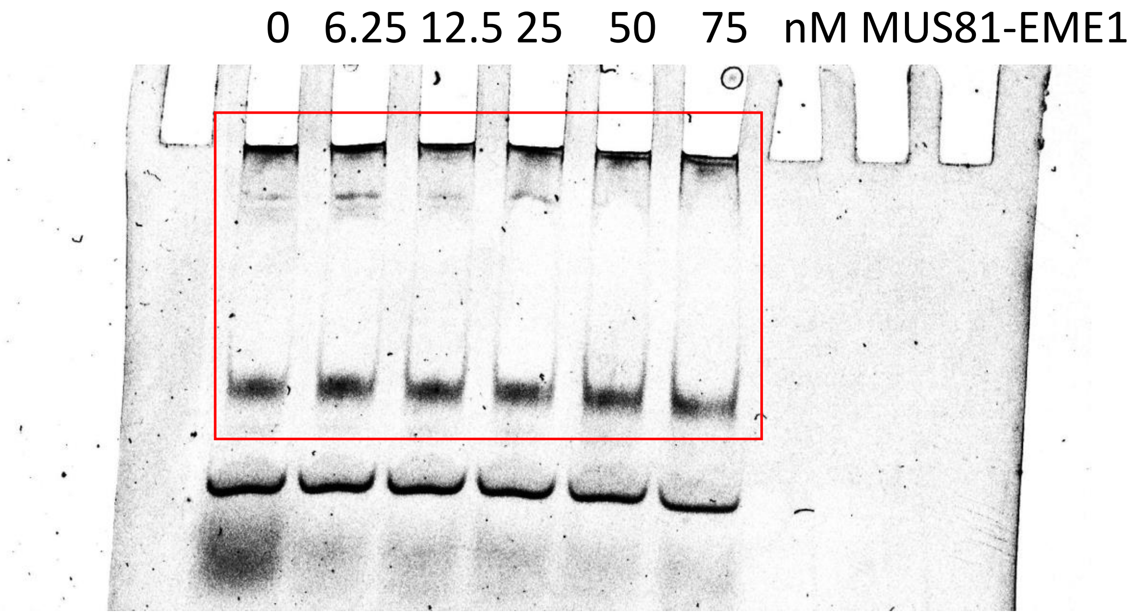

Figure 2B

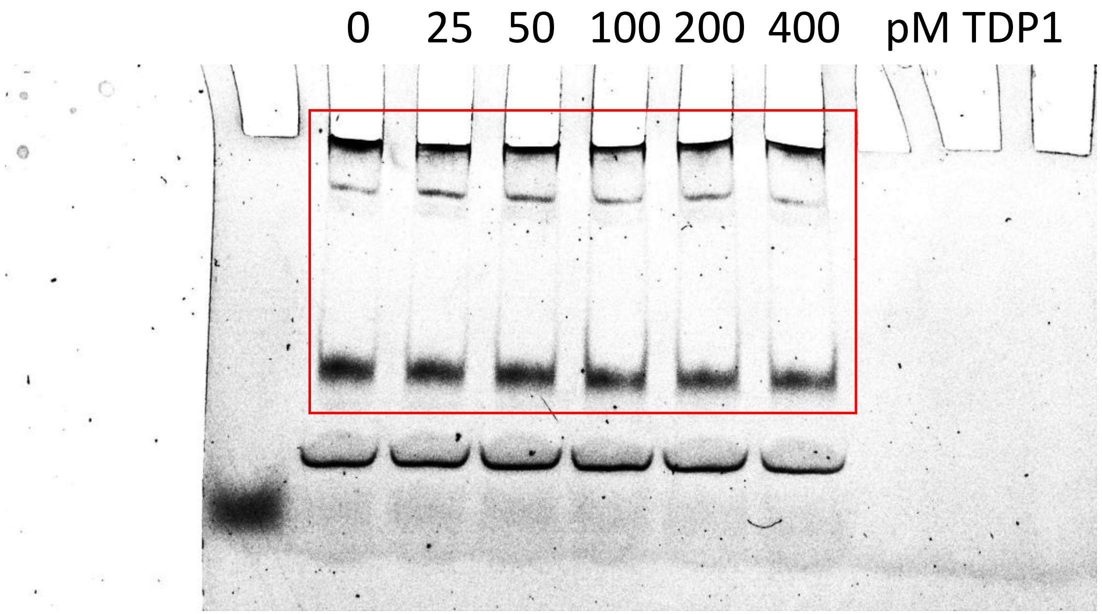

Figure 3A

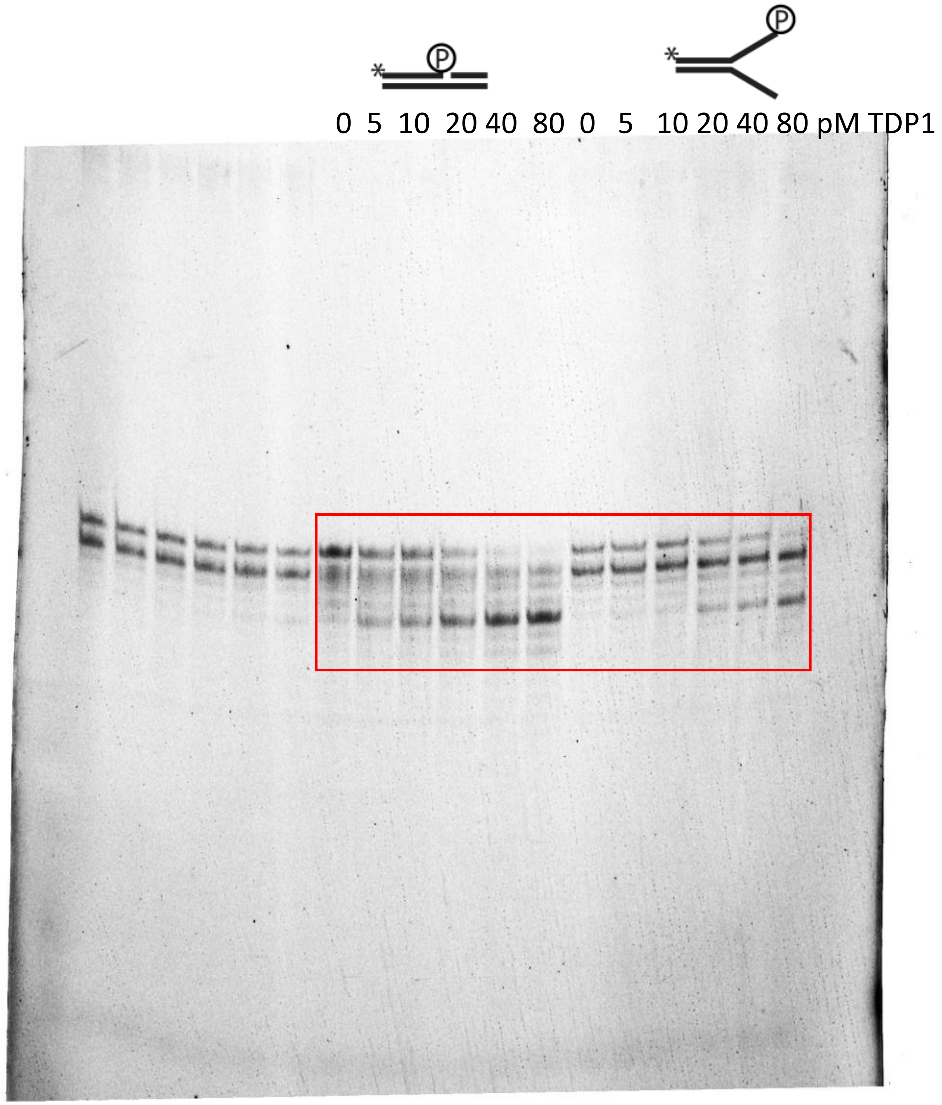

Figure 3A

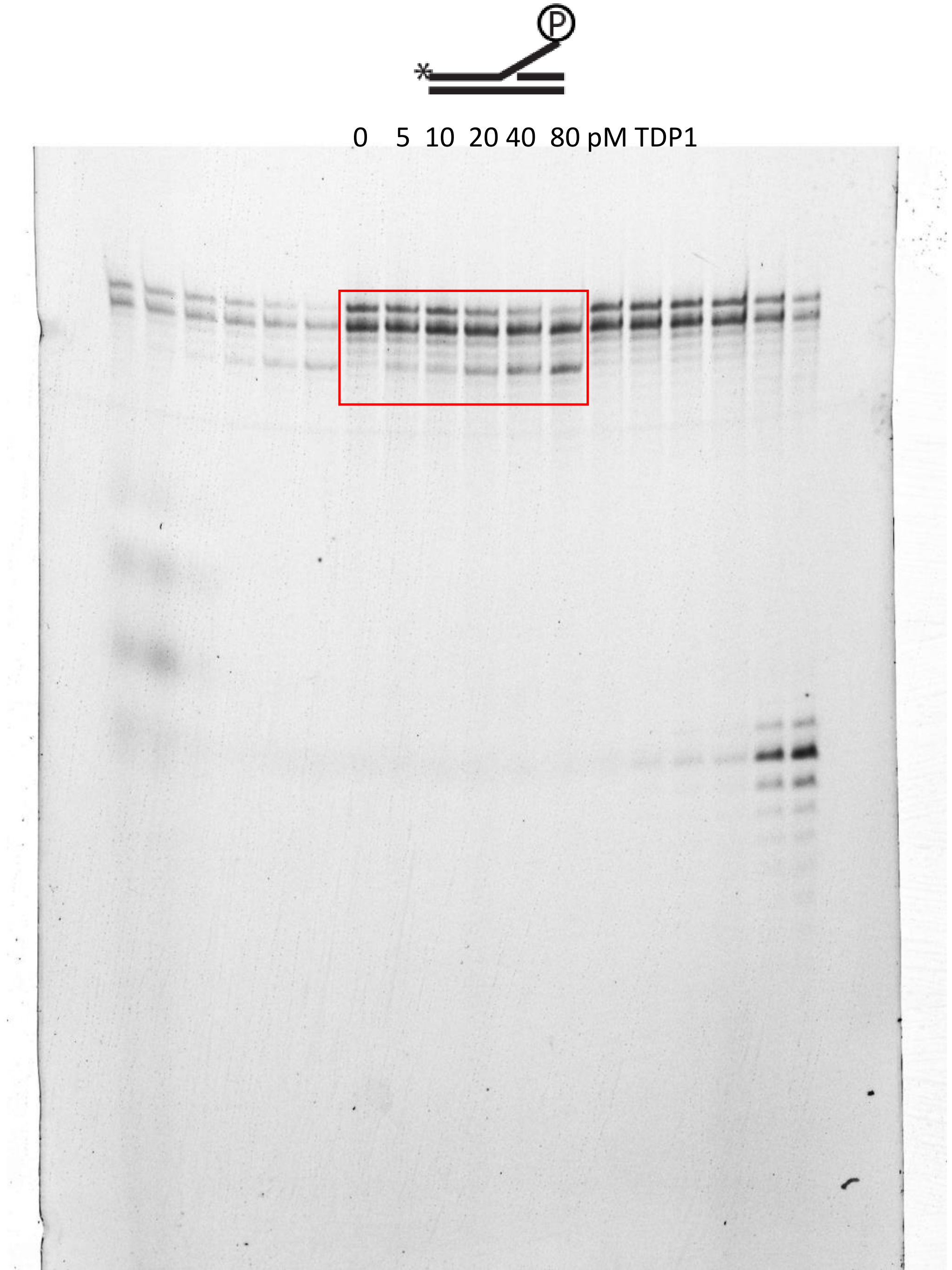

Figure 3B

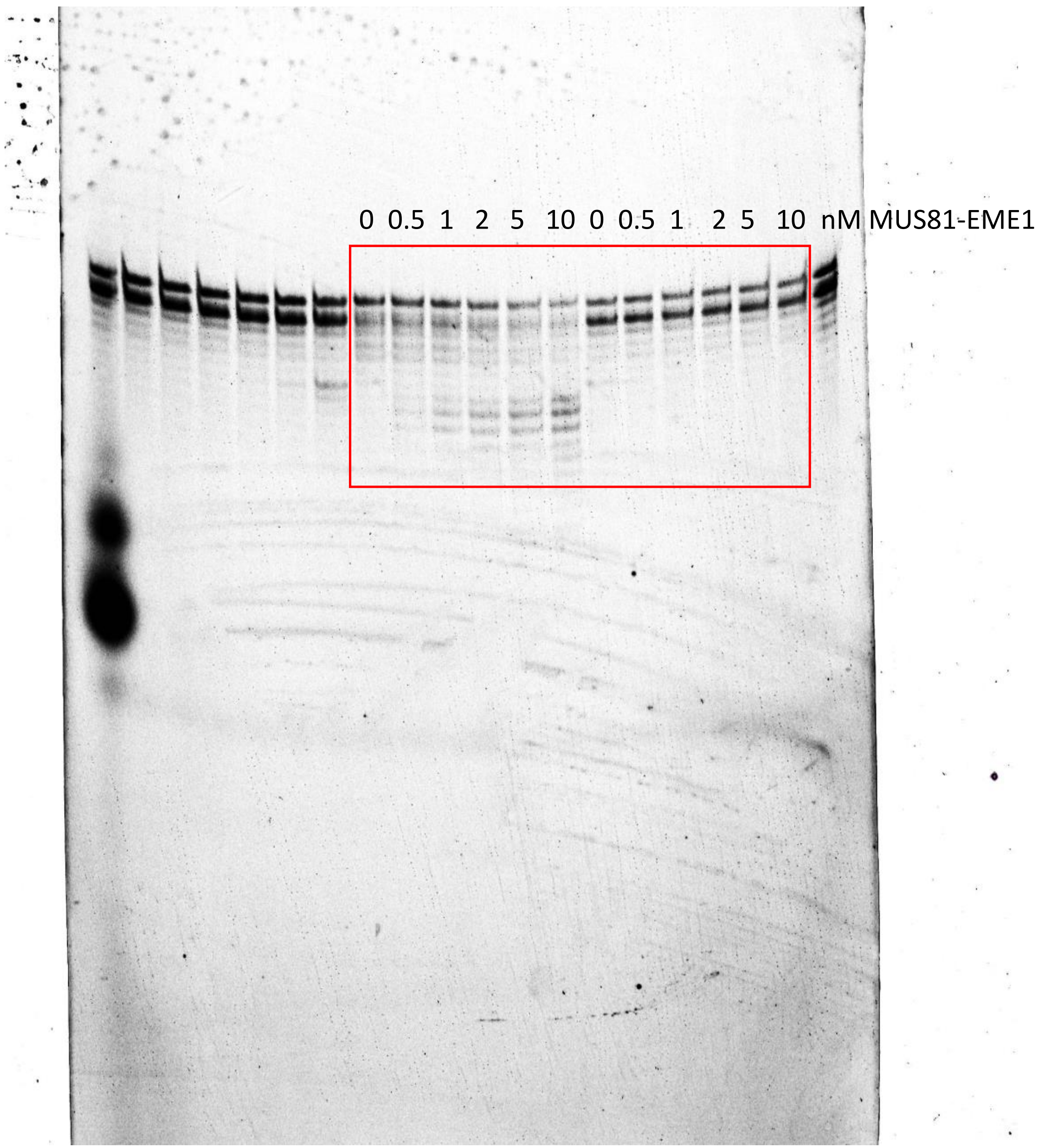

Figure 3C

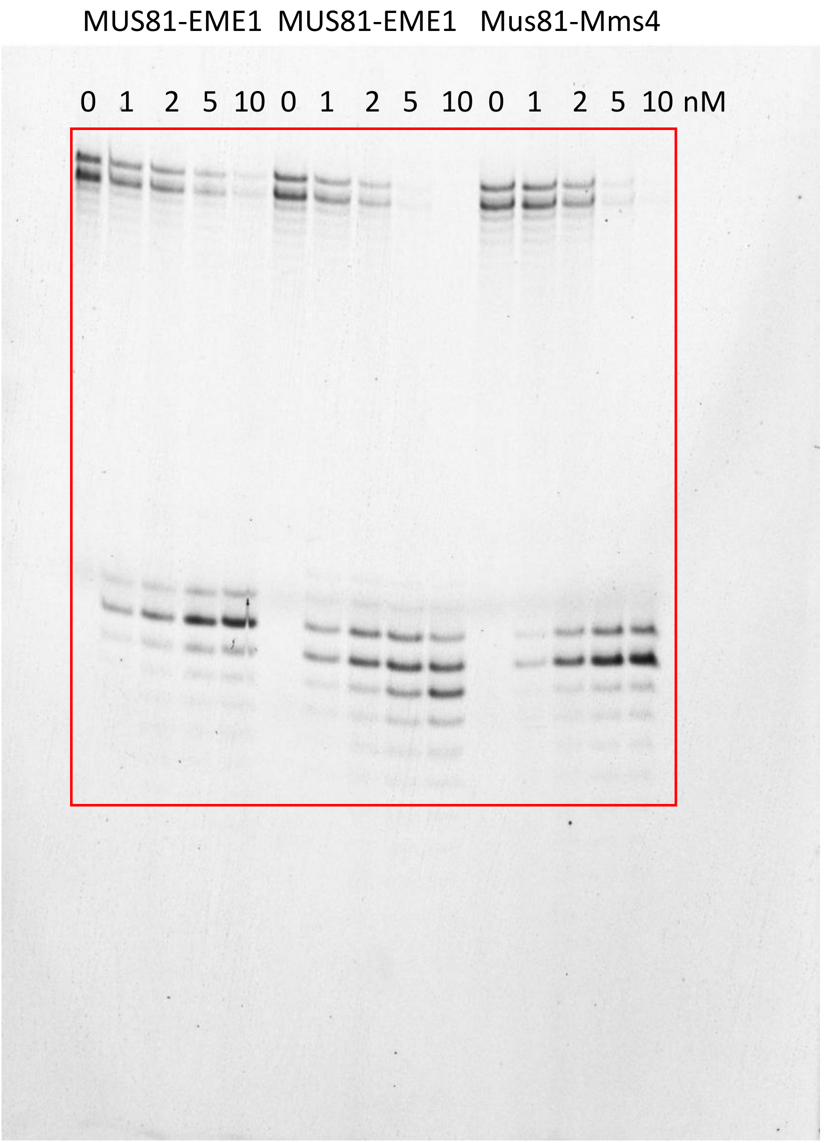

Figure 4A

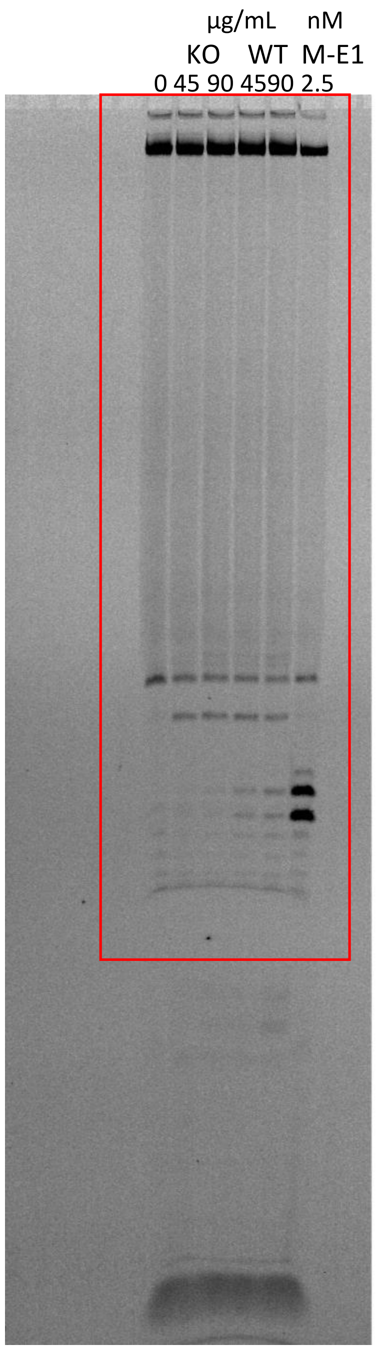

Figure 5A

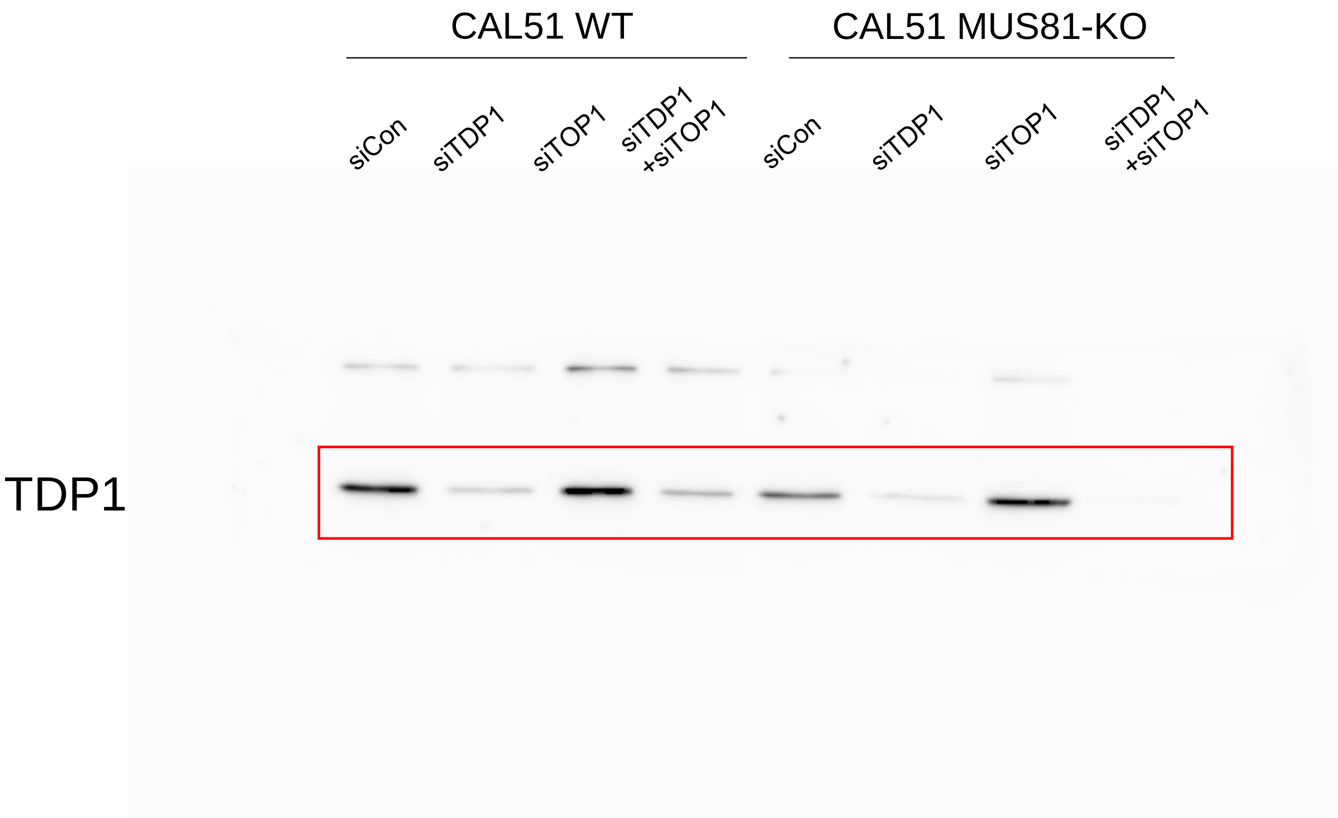

CAL51 WT
CAL51 MUS81-KO
siCon
siTDP1
siTOP1
siTDP1 +siTOP1
siCon
siTDP1
siTOP1
siTDP1 +siTOP1
TDP1

Figure 5A

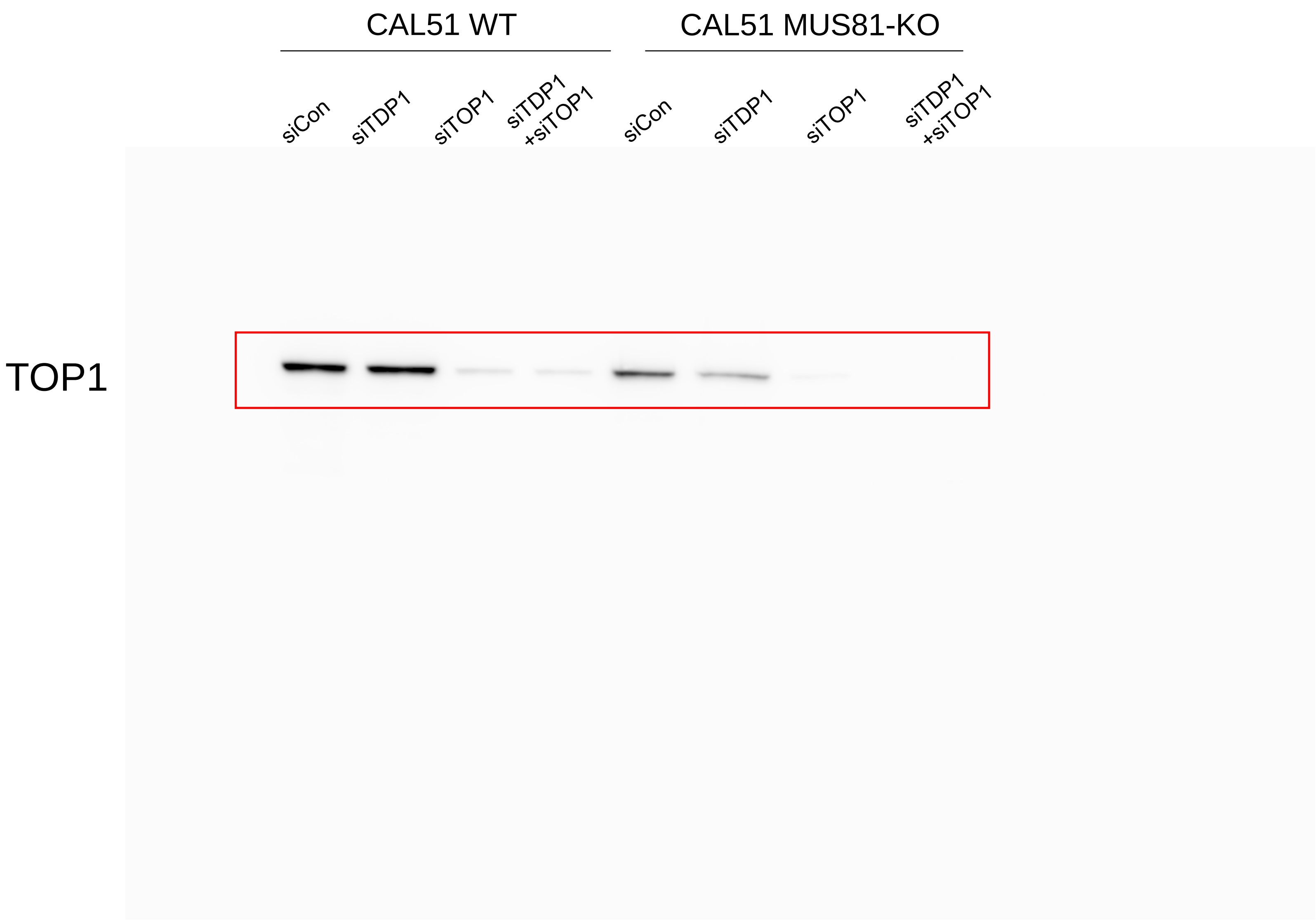
CAL51 WT
CAL51 MUS81-KO
siCon
siTDP1
siTOP1
siTDP1 +siTOP1
siCon
siTDP1
siTOP1
siTDP1 +siTOP1
TOP1

Figure 5A

Reblot after TOP1

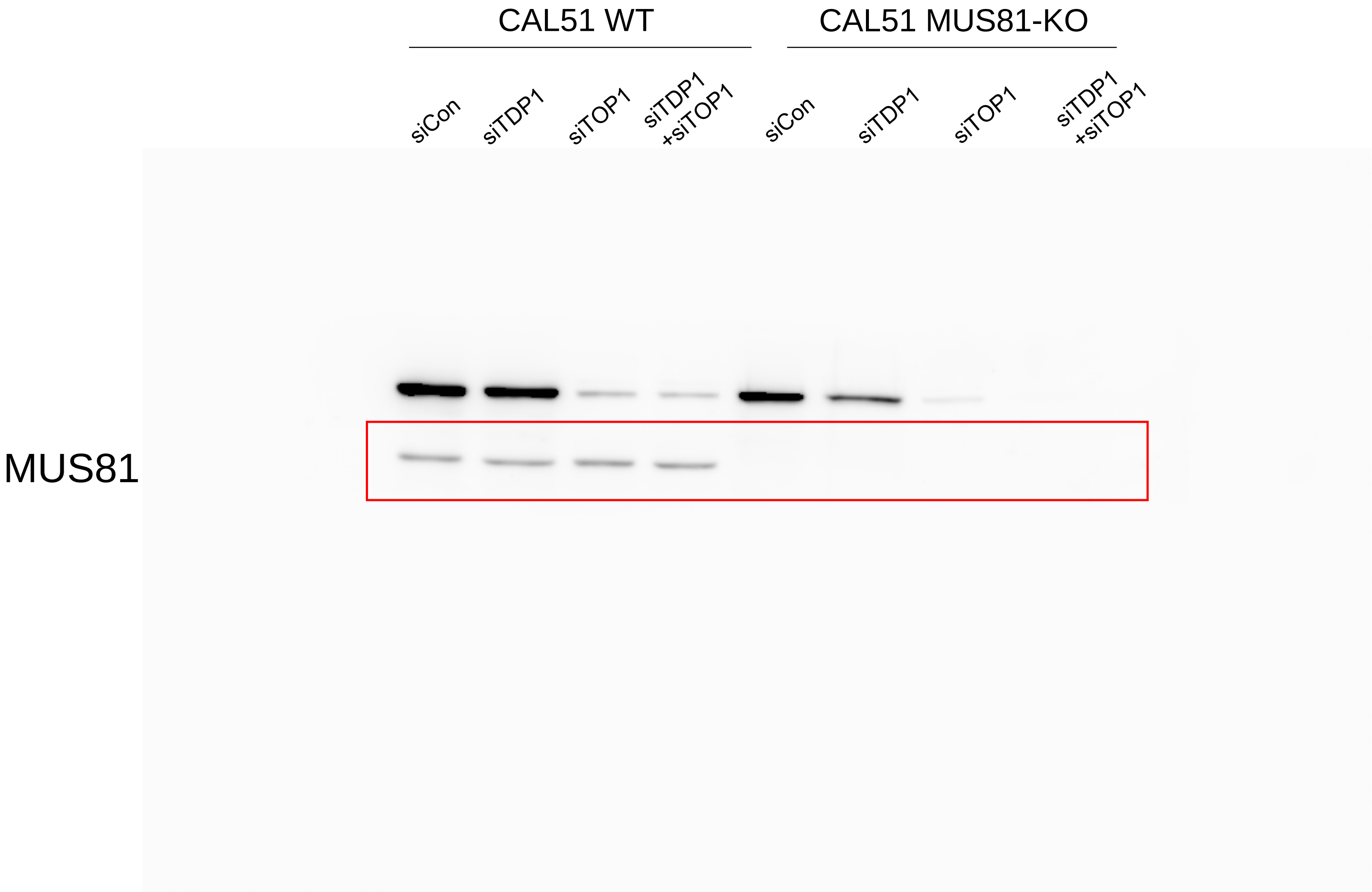

Figure 5A

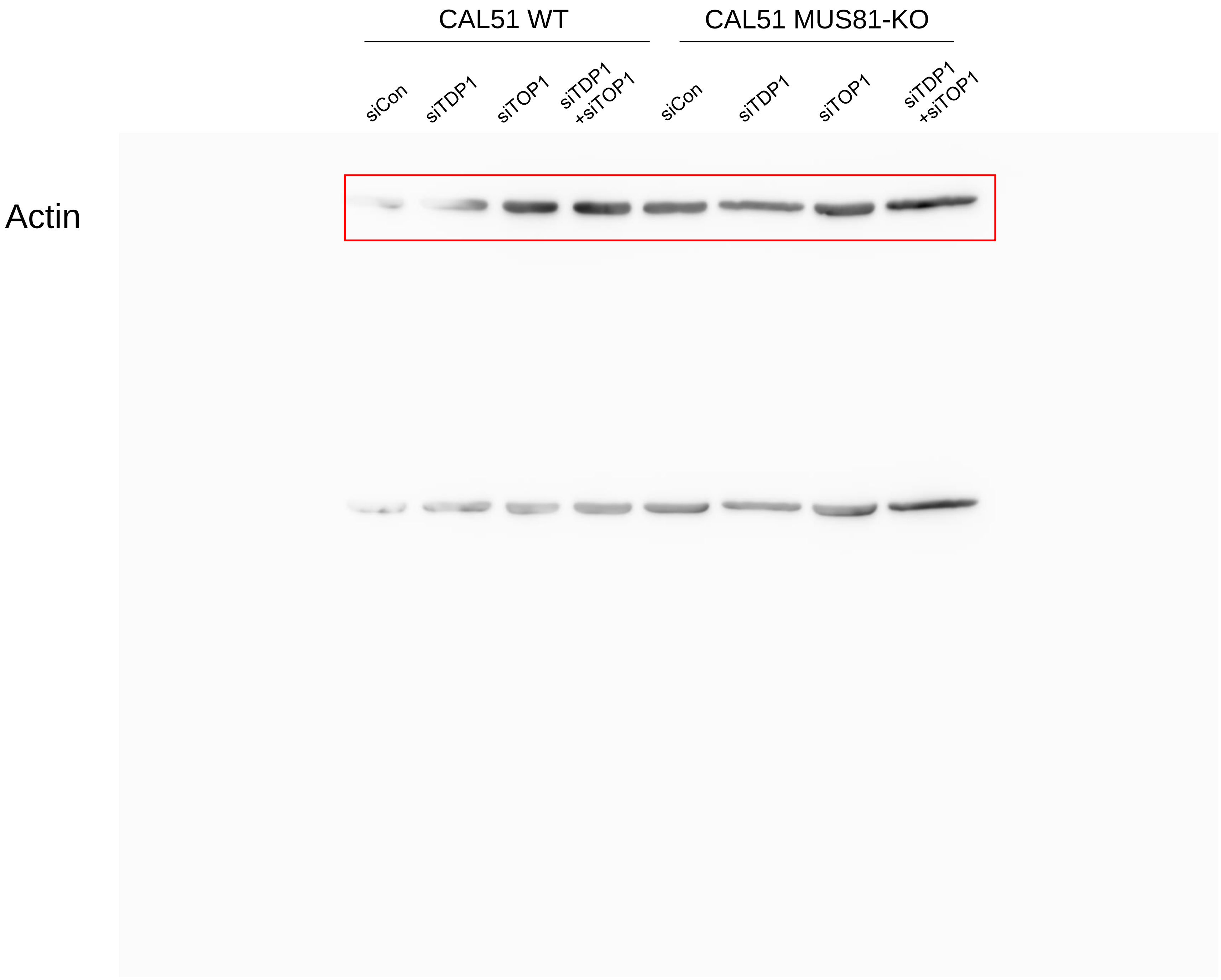

CAL51 WT
CAL51 MUS81-KO
siCon
siTDP1
siTOP1
siTDP1 +siTOP1
siCon
siTDP1
siTOP1
siTDP1 +siTOP1
Actin

Figure S2A

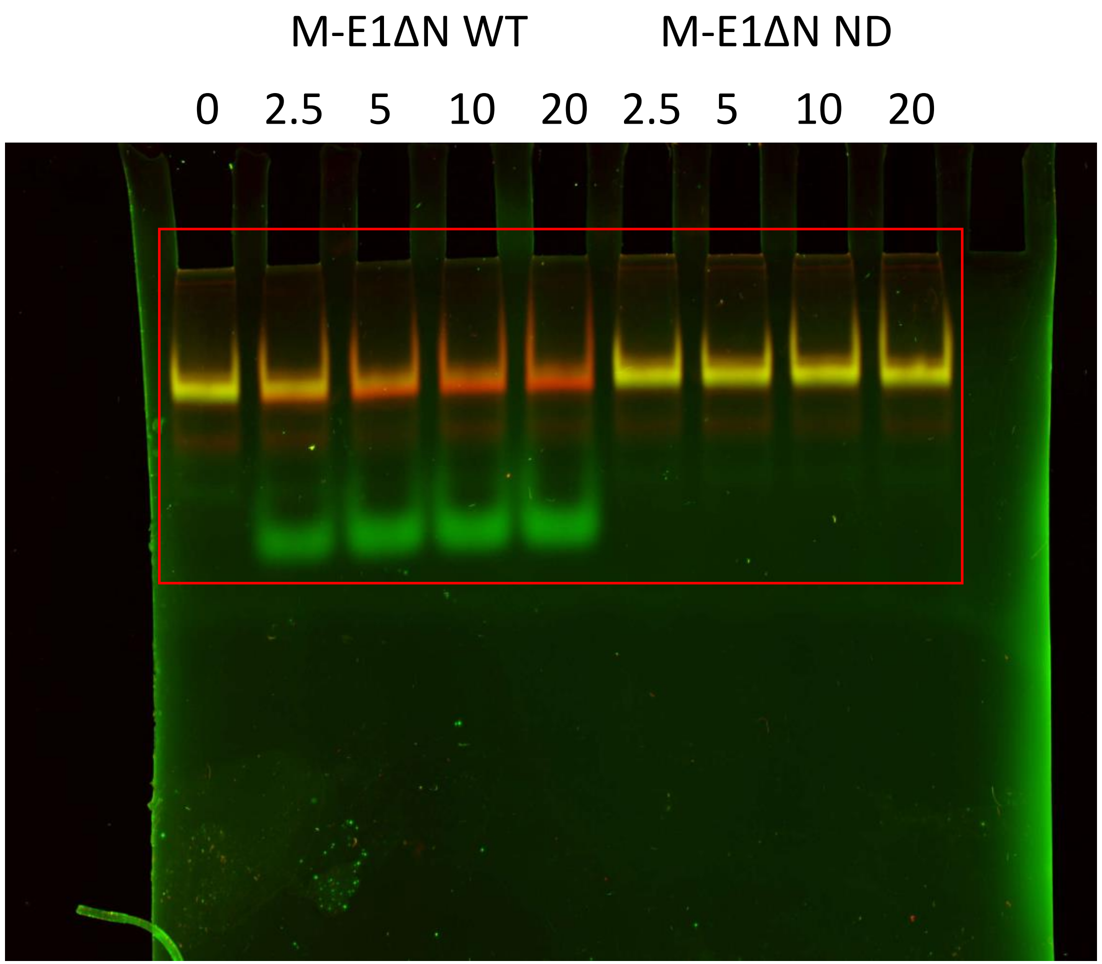

Figure S2B

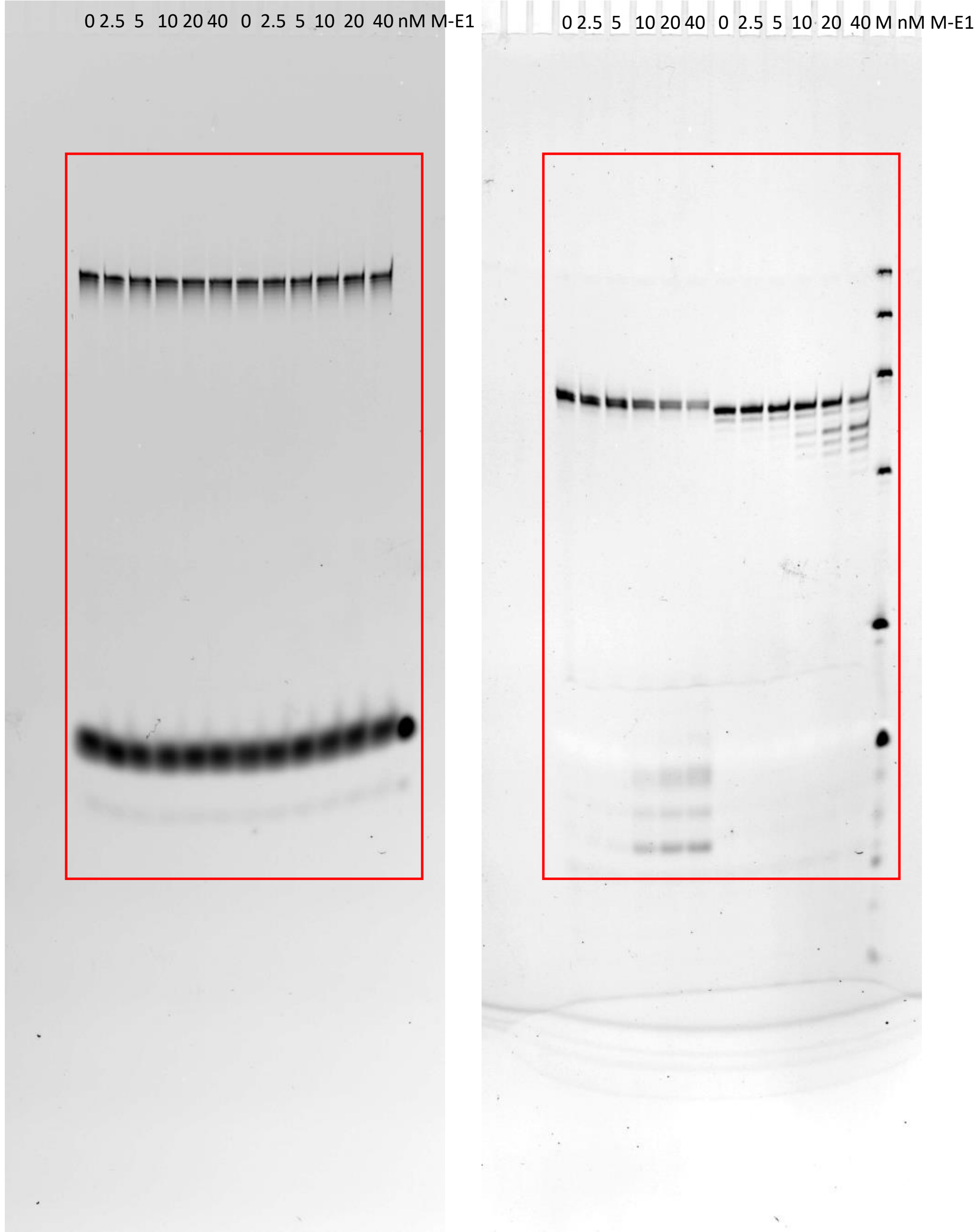
0 2.5 5 10 20 40 0 2.5 5 10 20 40 nM M-E1
0 2.5 5 10 20 40 0 2.5 5 10 20 40 M nM M-E1

Figure S3C

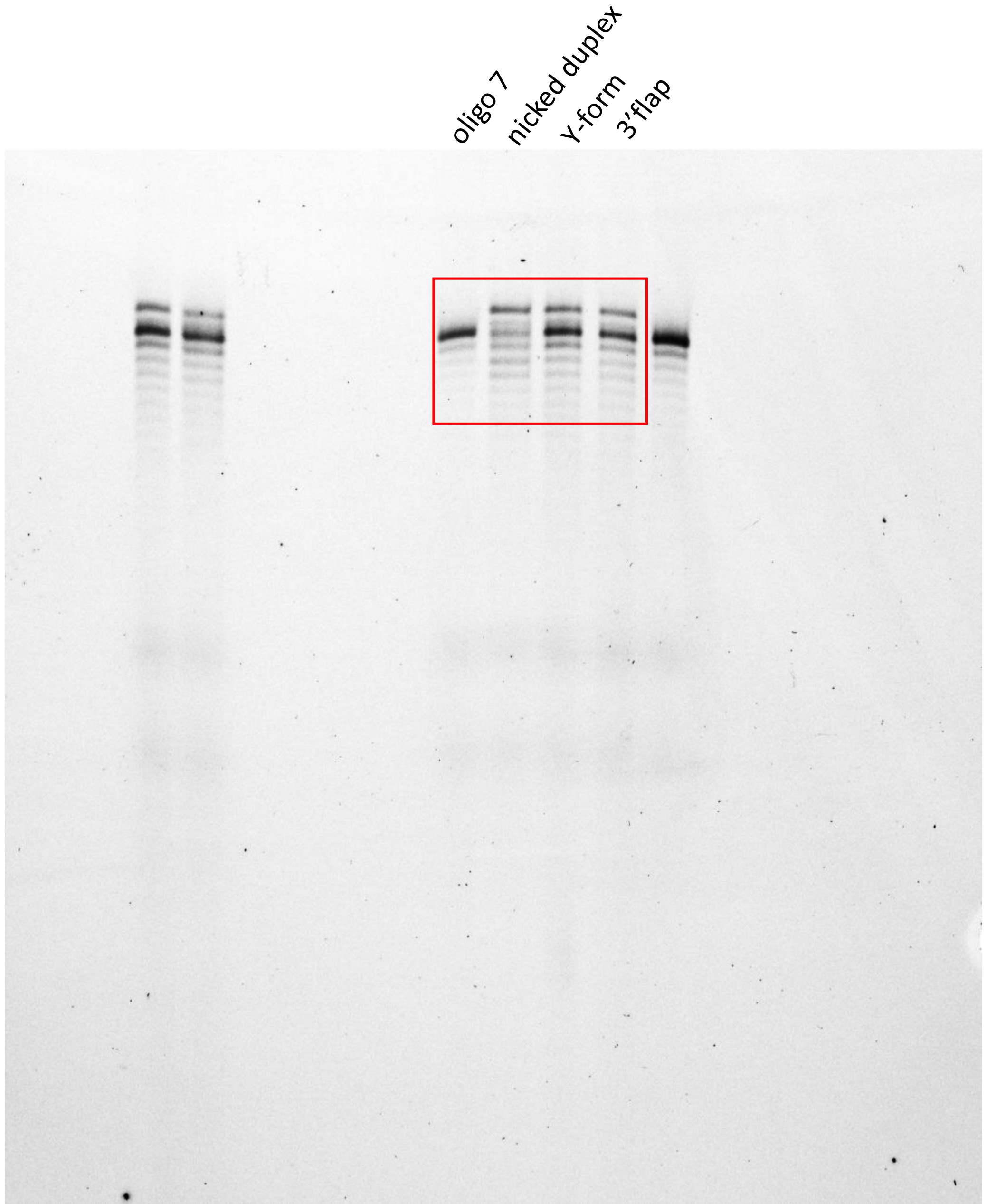

Figure S5

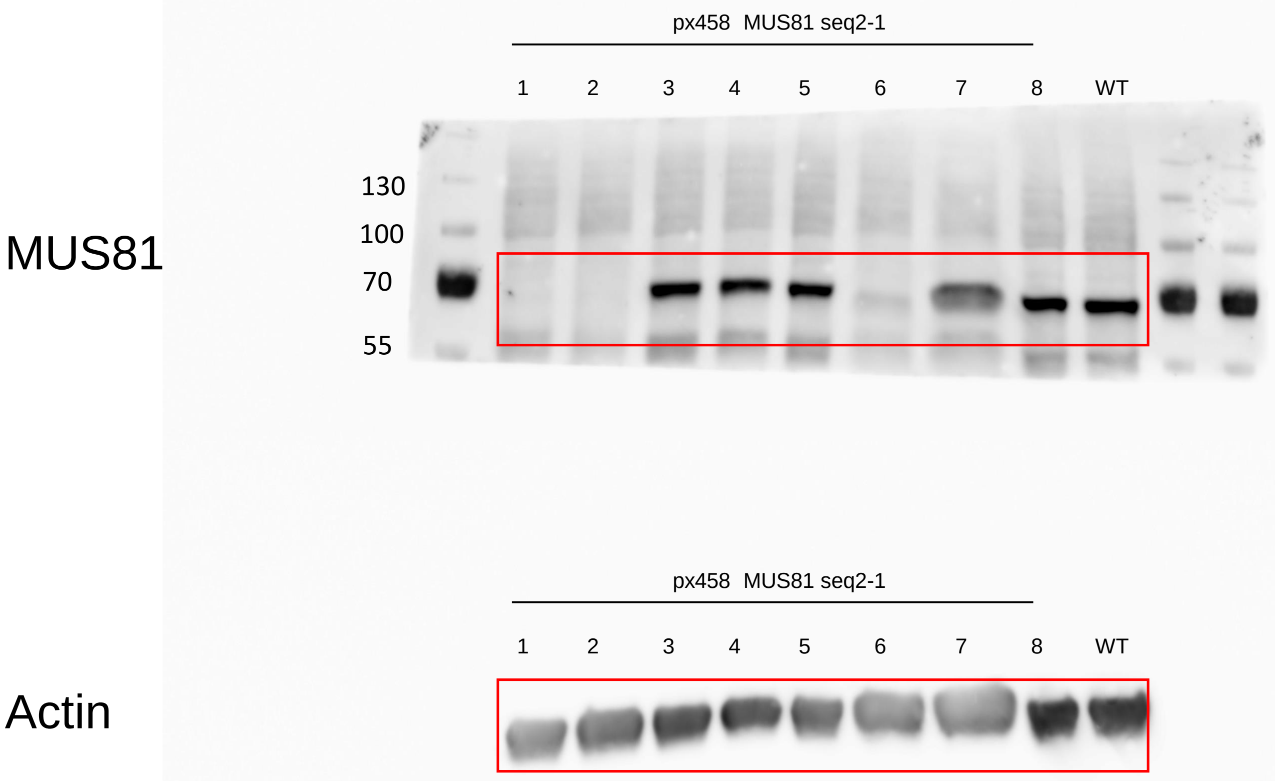
MUS81
Actin
px458 MUS81 seq2-1
1
2
3
4
5
6
7
8
WT
130
100
70
55
px458 MUS81 seq2-1
1
2
3
4
5
6
7
8
WT

Figure S6A

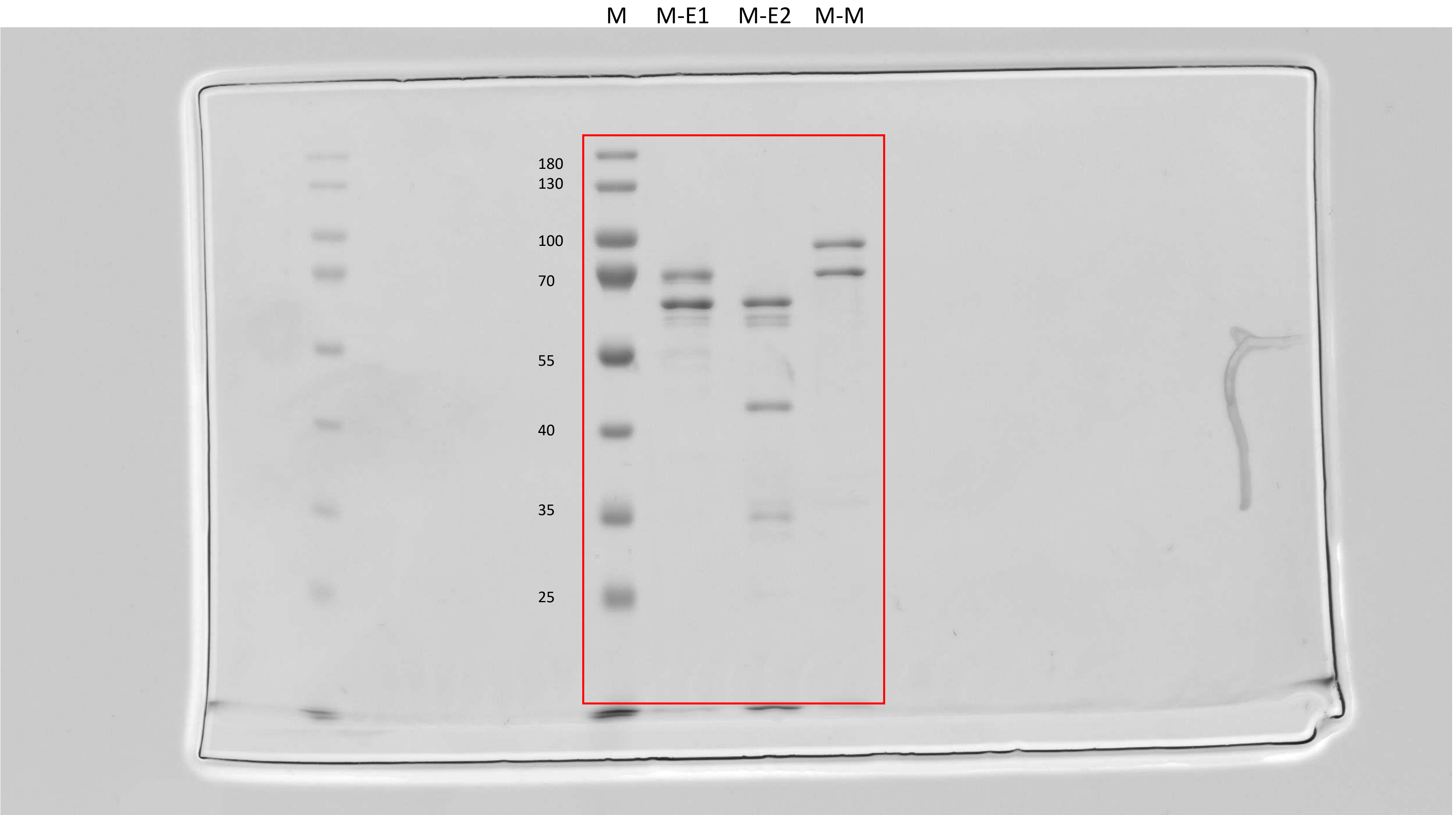

Figure S6B

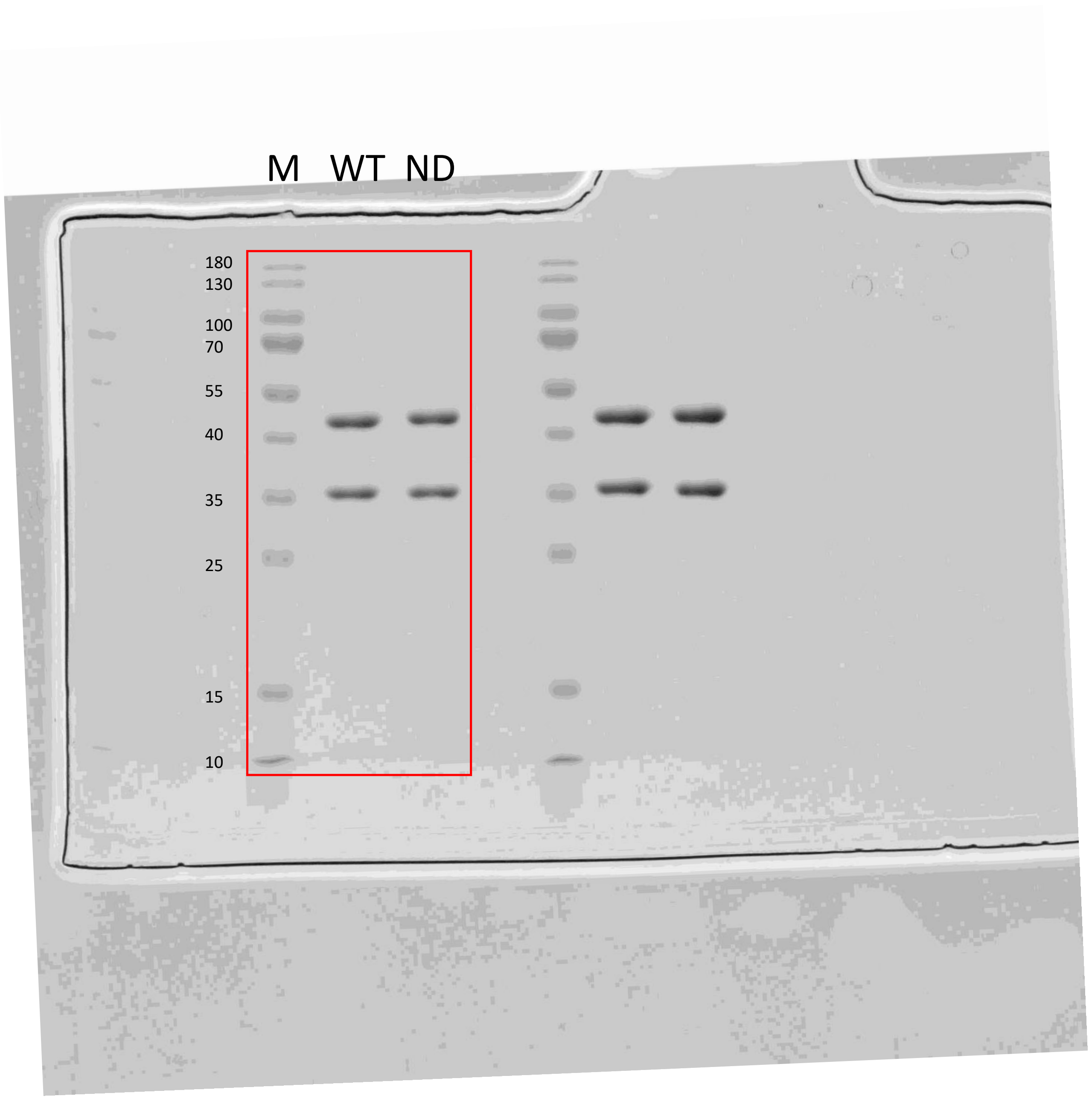

Supplement: Supplementary file 9 — Additional file 9. Uncropped images of gels and Western blots. Uncropped images of gels and Western blots shown in this paper. [file 12915_2023_1614_MOESM9_ESM.pdf]
